# CAR-T and CAR-NK as cellular cancer immunotherapy for solid tumors

**DOI:** 10.1038/s41423-024-01207-0

**Published:** 2024-08-12

**Authors:** Lei Peng, Giacomo Sferruzza, Luojia Yang, Liqun Zhou, Sidi Chen

**Affiliations:** 1 Department of Genetics, Yale University School of Medicine, New Haven, CT USA; 2 System Biology Institute, Yale University, West Haven, CT USA; 3 Combined Program in the Biological and Biomedical Sciences, Yale University, New Haven, CT USA; 4 Molecular Cell Biology, Genetics, and Development Program, Yale University, New Haven, CT USA; 5 Immunobiology Program, Yale University, New Haven, CT USA; 6 Yale Comprehensive Cancer Center, Yale University School of Medicine, New Haven, CT USA; 7 Department of Neurosurgery, Yale University School of Medicine, New Haven, CT USA; 8 Yale Stem Cell Center, Yale University School of Medicine, New Haven, CT USA; 9 Yale Liver Center, Yale University School of Medicine, New Haven, CT USA; 10 Yale Center for Biomedical Data Science, Yale University School of Medicine, New Haven, CT USA; 11 Yale Center for RNA Science and Medicine, Yale University School of Medicine, New Haven, CT USA

**Keywords:** CAR-T, CAR-NK, Cell therapy, Cancer immunotherapy, Solid tumor, Tumour immunology, Immunotherapy, Cancer therapy

## Abstract

In the past decade, chimeric antigen receptor (CAR)-T cell therapy has emerged as a promising immunotherapeutic approach for combating cancers, demonstrating remarkable efficacy in relapsed/refractory hematological malignancies in both pediatric and adult patients. CAR-natural killer (CAR-NK) cell complements CAR-T cell therapy by offering several distinct advantages. CAR-NK cells do not require HLA compatibility and exhibit low safety concerns. Moreover, CAR-NK cells are conducive to “off-the-shelf” therapeutics, providing significant logistic advantages over CAR-T cells. Both CAR-T and CAR-NK cells have shown consistent and promising results in hematological malignancies. However, their efficacy against solid tumors remains limited due to various obstacles including limited tumor trafficking and infiltration, as well as an immuno-suppressive tumor microenvironment. In this review, we discuss the recent advances and current challenges of CAR-T and CAR-NK cell immunotherapies, with a specific focus on the obstacles to their application in solid tumors. We also analyze in depth the advantages and drawbacks of CAR-NK cells compared to CAR-T cells and highlight CAR-NK CAR optimization. Finally, we explore future perspectives of these adoptive immunotherapies, highlighting the increasing contribution of cutting-edge biotechnological tools in shaping the next generation of cellular immunotherapy.

## Introduction

Immunotherapy, which aims to stimulate the immune system to eradicate cancer, has recently revolutionized cancer treatment and constitutes the fourth cornerstone of cancer therapy alongside surgery, radiation, and chemotherapy [1, 2]. Current cancer immunotherapy research encompasses a broad range of approaches, including antibodies, vaccines, cytokines, oncolytic viruses, bi-specific molecules, and cellular therapies [3, 4]. Among these, immune checkpoint inhibitors (ICIs) and adoptive cell therapy (ACT) have emerged as the most successful immunotherapy strategies for cancer treatment [5–9]. ICIs, which block immune checkpoints, have achieved significant tumor regression and changed standards of care for a variety of solid tumor malignancies, including melanoma, lung cancer, and head and neck cancer [5, 10, 11]. However, primary and acquired resistance remains common due to the scarcity of anti-tumor T cells and impaired memory T cells.

Unlike small molecule drugs or antibodies, cells have the potential to sense and dynamically respond to diseases [12]. Cellular immunotherapy, which involves the transfer of modified immune cells back into the patient, has rapidly grown in clinical investigation and achieved significant success in hematologic malignancies, though its use in solid tumor malignancies is still in its early stages [2, 13].

In this review, we explore the potential of CAR-T and CAR-NK cells, along with the current limitations of these treatment modalities against cancer, especially solid tumors. We present an overview of the engineering strategies implemented in recent years to address the main challenges limiting the CAR-T and CAR-NK cell effectiveness, showcasing the broad versatility of cellular immunotherapy. Finally, we highlight the potential advantages and limitations of CAR-NK cells over CAR-T cells and outline the future perspectives of these cancer therapies.

## CAR-T cell cancer therapy

T cells, as a key component of the adaptive immune system, can be divided into different subgroups based on function, co-receptor expression (CD4 or CD8), trafficking, metabolism, and lifespan, playing a crucial role in fighting cancer due to their potent ability to recognize and eliminate cancerous cells [14]. Consequently, T cells have emerged as primary candidates for cancer immunotherapy. However, in immunocompetent patients, tumors often undergo “cancer immunoediting”, enabling them to evade cellular immunity and establish a microenvironment that facilitates tumor outgrowth [15]. Therefore, effective ACT needs to overcome the tumor immune escape mechanisms, including T cell dysfunction [16–18] and the absence of tumor-associated antigens (TAA), such as lineage antigens in hematological malignancies. This can be achieved by expanding and activating T cells ex vivo or genetically engineering them to boost their cancer-fighting capabilities.

Currently, there are three major modalities of autologous T cell immunotherapy: tumor-infiltrating lymphocyte (TIL) therapy, genetically engineered T cell receptors T (TCR-T) cell therapy, and CAR-T cell therapy [19]. TIL therapy, which involves expanding a heterogeneous population of endogenous T cells with a pool of native TCRs from a harvested tumor, has been approved by the FDA for the treatment of advanced melanoma (FDA news release on 2/16/2024). This strategy is particularly promising for “hot tumors”, characterized by a tumor microenvironment (TME) enriched with TILs, indicating a preexisting immune response [20]. TCR-T cell therapy involves expanding T cells with genetically encoded TCRs directed toward specific antigen targets. In contrast, CAR-T cell therapy involves expanding genetically engineered T cells equipped with synthetic receptors (CARs) that recognize specific antigens on cancer cells [21]. Additionally, more sophisticated engineered T cells, such as synthetic TCR and antigen receptor (STAR-T) [22] and HLA-independent T cell receptors (HIT) [23], are also on the horizon.

**Fig. 1:**
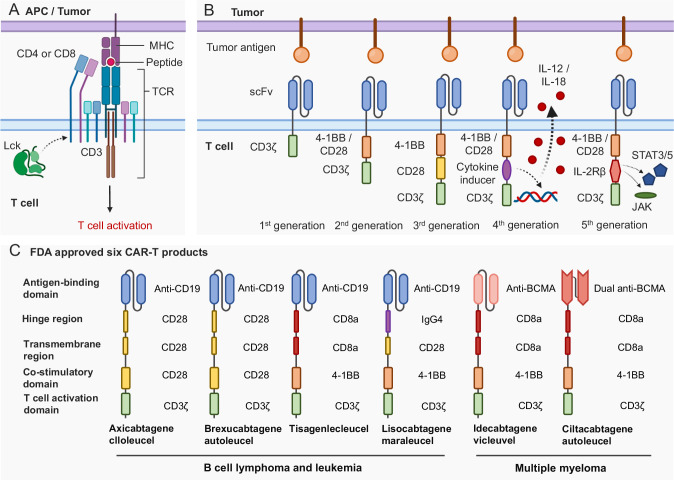
Chimeric antigen receptor (CAR) construct. **A** When a T cell encounters antigen-presenting cells or tumor cells, T cell receptor (TCR) and CD3 complex, together with CD4 or CD8, will recognize MHC complex-presented peptides, then trigger TCR signaling cascade, leading to T cell activation. **B** The first-generation CAR majorly involves two parts: scFv at the extracellular part and CD3ζ chain at the intracellular part. ScFv domain can recognize tumor surface antigens, and then the CD3ζ chain will directly activate T cells. The second-generation CAR incorporated a co-stimulation domain, either 4-1BB or CD28 signaling domain. The third-generation CAR has both 4-1BB and CD28 signaling domains. **C** Differences of antigen-binding domain, hinge region, transmembrane region, co-stimulatory domain, and T cell activation domain of six FDA-approved CAR-T products. Those products are used to treat B-cell lymphoma, B-cell leukemia, and multiple myeloma. APC antigen-presenting cell, MHC major histocompatibility complex, TCR-T cell receptor, scFv single-chain variable fragment

The recognition and killing of cancer cells by T cells rely on canonical TCR signaling. Initially, T cells are primed by peptide antigens presented by major histocompatibility complex (MHC), which activates a series of downstream signals to facilitate cancer killing [16] (Fig. 1A). In contrast, CARs were first proposed and invented by Eshhar et al. to enable T cell cytotoxicity independent of MHC restriction, allowing for target-specific cytotoxicity and broader therapeutic applicability [24]. The first generation of CARs consists of an extracellular single-chain variable fragment (scFv) that recognizes specific antigens, linked via a hinge and transmembrane (TM) domain to a C-terminal CD3ζ activation domain (Fig. 1B). These antibody-based antigen receptors redirect the inherent cytotoxic nature of T cells to kill cancer cells in an antigen-specific manner.

However, the first generation of CAR-T cells, which relied solely on the CD3 ζ-chain to simulate TCR signaling, exhibited limited proliferation, engraftment, and cytotoxicity, resulting in unsatisfactory clinical efficacy. Subsequent modifications to CAR molecules led to the development of second and third-generation CAR-T cells, which included one (second-generation) or two (third-generation) intracellular co-stimulatory (ICOS) domains upstream of CD3ζ (Fig. 1B) [25]. These enhancements improved CAR-T cell cytotoxicity, cytokine production, and proliferation in response to target stimulation.

Currently, there are six FDA-approved CAR agents targeting CD19 or BCMA for hematological malignancies [26]. All approved CAR products employ a second-generation CAR construct, consisting of an antigen-binding domain, a hinge region, a TM region, a co-stimulatory domain (CD28 or 4-1BB), and a T cell activation domain (Fig. 1C). These CAR-T cell therapies have achieved durable remission in many patients with hematological malignancies. In pivotal trials, CD19-CAR therapy outperformed the standard of care (SOC) as a second-line treatment for large B-cell lymphoma and has shown high effectiveness as a first-line therapy. BCMA-CAR therapy has demonstrated high response rates in multiple myeloma [27]. Recently, ongoing clinical trials conducted by major manufacturer pharmaceutical companies are comparing the efficacy of CAR-T cells to SOCs, aiming to introduce them to earlier lines of treatment for multiple myeloma. Such success has ushered in the era of cell therapy for hematological diseases.

### Challenges in CAR-T cell therapy for solid tumors

Substantial attention and efforts have been devoted in recent years to improve CAR-T cell therapy for solid tumors. As of June 2024, data from ClinicalTrials.gov indicate a total number of 405 CAR-T cell clinical trials targeting solid tumors with varying statuses (29 completed, 30 active/not recruiting, 179 actively recruiting or enrolling by invitation, 90 with unknown status, 39 suspended/terminated/withdrawn, and 38 not yet recruiting). Despite showing signs of activity in solid tumors, outcomes from clinical trials have been disappointing to date, with no consistently high rates of durable responses observed [26].

The exact reasons why CAR-T cells have underperformed in solid tumors remain unclear, primarily due to insufficient biological information to evaluate key aspects of therapy. Most clinical studies evaluating CAR-T cells in solid tumors have predominantly reported generic tumor response parameters and the presence/persistence of CAR-T cells in peripheral blood (PB) [28, 29]. However, pivotal details regarding CAR-T cell infiltration, phenotype, and interactions with the TME are largely lacking, as they require post-infusion bioptic data.

As an illustrative example, the application of CAR-T cells in patients affected by high-grade glioma can be cited. Currently, eleven single report or Phase I clinical trials have been performed [30–40], including ~130 patients. However, post-infusion pathological data have only been obtained for 11 patients, always following clinical-driven surgical indications or post-mortem. These data suggest several important findings: (1) CAR-T cells patchy infiltrate the tumor when administrated intravenously [38], (2) their persistence appears to be limited [30, 31, 38], and (3) the therapy administration is followed by an upregulation of immuno-suppressive signals in TME [38]. Furthermore, both pathological data and liquid biopsy samples acquired in these trials have highlighted the well-known ability of glioblastoma in antigen escaping. However, the non-systematic acquisition of the pathological data precludes speculation on the mechanistic series events that allow tumor immunoescape [41].

**Fig. 2:**
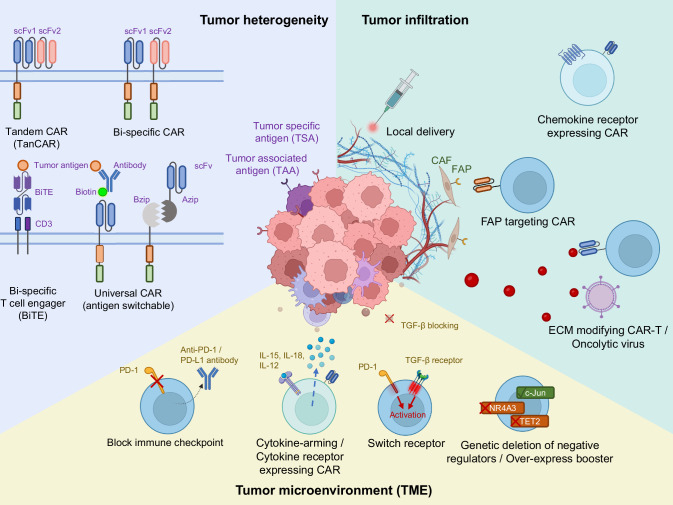
Challenges of CAR-T faces and engineering strategies to overcome solid tumor. Treating solid tumors poses three major challenges for CAR-T cells: tumor heterogeneity, tumor infiltration, and inhibitory tumor microenvironment (TME). There are two types of tumor antigens, tumor-specific antigen (TSA) which only expresses in tumor cells, and tumor-associated antigen (TAA) which can be expressed in normal cells as well. Meanwhile, tumor cells may express tumor antigens at different levels as well. To overcome heterogeneity, efforts have been made to engineer CARs with two different scFvs (TanCAR) or express two different CARs recognizing different antigens (Bi-specific CAR). Bi-specific T cell engager can link tumor antigen and CD3 complex to initiate T cell tumor killing. Solid tumor also has physical barriers and extracellular matrix that limit CAR-T cell infiltration. To overcome this challenge, researchers have tried local delivery of CAR-T cells, engineering chemokine receptor-expressing CAR, FAP-targeting CAR, or EMC modifying CAR-T or oncolytic virus. Immune suppressive TME is another big challenge which consists of inhibitory immune cell infiltration, immune suppressive factors such as TGF-β, as well as chemical environment alterations. Researchers have tried to block immune checkpoints such as PD-1, using genetic deletion, administration of anti-PD-1/PD-L1 antibodies, or T cell autocrine antibodies. Efforts have also been made to engineer cytokine-arming or cytokine receptor-expressing CAR, replace inhibitory domains with activation domains in PD-1 or TGF-β as switch receptor, and delete T cell internal negative regulators or over-express T cell function boosters

Without this, we rely solely on reasonable considerations obtained from solid tumor biology or inferences coming from mouse models. In fact, solid cancers present unique challenges for CAR-T therapy. First, unlike B-cell malignancies, which possess several lineage-specific epitopes, cells within most of the solid tumors are heterogeneous [42–44]. Additionally, even when a tumor antigen is shared with healthy cells, in hematological tumors it can be feasible to sacrifice the entire antigen-positive lineage to eradicate the cancer. This is because the intrinsic characteristic of the bone marrow tissue, that can be ablated, cytoreducted, stimulated with growth factors, and even transplanted allowing the survival of the patient. In contrast, most solid tumors originate from tissues where a widespread autoimmune cross-reaction would be life-threatening. For this reason, identifying tumor-specific antigens that can be targeted by CAR-T cells has proven difficult. Although antigens that are overexpressed in tumors or TAA have been chosen, there are non-negligible levels of antigen expression in healthy tissues, raising concerns about toxicity issues [43, 45]. Second, fibrotic tumor stroma of solid tumor, which is comprised of extracellular matrix (ECM) and cancer-associated fibroblasts (CAFs), along with the abnormal vasculature at the tumor site, create physical barriers that impede CAR-T cell infiltration and penetration into the tumor [46–48]. Additionally, the immuno-suppressive TME of solid tumor characterized by numerous suppressive immune cells such as regulatory T cells (Tregs) and myeloid-derived suppressor cells (MDSCs), as well as immuno-suppressive ligands and agents such as programmed cell death 1 ligand 1 (PD-L1), TGF-β, and adenosine, further hinder CAR-T cell cytotoxic activity, proliferation, and persistence in combating solid tumors [43, 49] (Fig. 2).

### Engineering strategies to improve CAR-T cell therapy for solid tumor

#### Combating antigen heterogeneity in solid tumor

Numerous strategies have been explored to address the antigen heterogeneity of solid tumors with the primary focus on achieving multi-target targeting (Fig. 2). One approach involves combinatorial strategies, such as sequential or combination treatments involving different CAR-T cell products. By concurrently targeting multiple antigens, this strategy aims to mitigate antigen escape phenomena commonly associated with CAR-T cell therapy. This strategy has already been demonstrated to be clinically safe and feasible in diffuse large B-cell lymphoma [50, 51] and may represent a promising approach also in solid tumors. Another innovative avenue is the development of multi-target CAR-T cells, achieved through the incorporation of two distinct CAR constructs into T cells or the utilization of bi-specific or Tandem (TanCAR) CAR-T cells. For instance, Muhammad et al. synthesized a novel TanCAR, comprising a tandem arrangement of IL13 and EphA2 single-chain variable fragments (scFv), demonstrating that the IL13-anti-EphA2 TanCAR exhibited markedly stronger anti-tumor efficacy compared to single CAR-T cells, both in vitro and in vivo [52].

Additional modification of CAR-T cells has been explored to enhance their targeting capabilities. One such strategy involves enabling CAR-T cells to secrete bi-specific T cell engagers (BiTEs) which engage with additional targets, thereby augmenting their anti-tumor efficacy. For example, Wing et al. designed folate receptor alpha (FR-α) targeting T cell engager CAR-T cells (anti-EGFR BiTEs) and showed that anti-EGFR BiTEs increased the anti-tumor efficacy of anti-EGFR CAR-T cells in a mouse model of glioblastoma [53]. Moreover, in a recent clinical trial, CARv3-TEAM-E T cells were evaluated in glioblastoma expressing the epidermal growth factor receptor variant III (EGFRv3). These cells were designed to recognize both this tumor-specific antigen, as well as the wild-type EGFR protein, through the secretion of a T cell-engaging antibody molecule (TEAM) [40]. In this way, TEAM-E T cells provide a potential solution to overcome the loss of EGFR variant III observed in glioblastoma after target immunotherapies like peptide vaccination [54] or CAR-T cells [38]. This Phase 1 trial has shown the feasibility and relative safety of the intracranial injection of the TEAM-E T cells. All of the three patients enrolled in the trial have shown a promising initial radiological response, albeit transient.

Furthermore, universal CARs are developed to realize multiple antigen targeting. Adapter elements serve as key components to enable the targeting of multiple antigens with a common CAR-T cell population. Noteworthy examples include avidin-linked CAR-T cells, leveraging the binding affinity between avidin and biotin to recognize multiple targets [55]. Similarly, approaches involving the engineering of CAR-T cells with Fcγ receptors, enabling recognition of the common Fc domain of antibodies, have been investigated to achieve multi-antigen targeting [56]. These strategies represent promising advancements in the field of CAR-T cell therapy, offering innovative solutions to overcome challenges associated with tumor heterogeneity and antigen escape.

Lastly, CAR-T cell therapy can be combined with vaccination against the target antigen recognized by the CAR. Once expressed by antigen-presenting cells, the target is able not only to promote CAR-T cell expansion and maturation in a memory phenotype [57] but also to engage the host immune cells to mount a response against different antigens of the same target [58].

This mechanism, known as epitope spreading, confers robustness to the immunotherapy while simultaneously addressing pre-treatment antigen heterogeneity and antigen loss induced by the treatment. Evaluated in Phase I/II clinical trial in advanced solid tumors, the mRNA CAR-T cell-amplifying vaccine showed no alteration of the safety profile of the CLDN-6 specific CAR-T cells [59]. Furthermore, despite this first publication being incomplete due to an amendment to the protocol, this trial has shown encouraging results in terms of disease control in a group of refractory tumors.

#### Increasing trafficking and infiltration into solid tumors

To combat cancer cells, CAR-T cells are requisite to contact and stimulated by its cognate antigens on tumors. Leveraging various cytokine and chemokine receptors capable of mediating immune cell trafficking, researchers have endeavored to engineer CAR-T cells to improve their trafficking and infiltration into solid tumors [60, 61] (Fig. 2). Jin et al. discovered significant amounts of IL-8, CXCL1, and CXCL2 were released from tumors. They engineered CARs modified with IL-8 receptors, CXCR1, and CXCR2, demonstrating that these modified CARs could be guided to migrate into tumors, inducing an enhanced anti-tumor response in solid tumors [62]. Moreover, the activation of CC-chemokine ligand 17 (CCL17) and CCL22, secreted by Reed–Sternberg cells of Hodgkin lymphoma (HL), activated their receptor CC-chemokine receptor 4 (CCR4) expressed T helper cells and Tregs [63]. Savoldo’s group showed that CD30 CAR-T cells co-expressing CCR4 (CCR4.CD30.CAR-T cells) exhibited improved tumor homing and anti-lymphoma activity compared to CD30 CAR-T cells lacking CCR4 expression [64]. Later on, data from a clinical trial (NCT03602157) further showed CCR4.CD30. CAR-T cells’ promising efficacy in patients with relapsed/refractory (r/r) classical HL, and displayed enhanced trafficking to the skin, rendering them more effective in CD30+ cutaneous T cell lymphomas [65]. Similarly, modifying CAR-T cells with the CCL2 cognate receptor CCR2b resulted in a 10-fold increase in infiltration of anti-GD2 CAR-T cells into neuroblastoma xenograft tumors and a 12-fold increase in infiltration into mesothelioma xenografts compared to unmodified CAR-T cells [66, 67]. Li et al. enhanced CAR-T cells by co-expressing IL-7 and CCR2b, demonstrating improved CAR-T cell survival and infiltration in glioblastoma and melanoma [68]. CCR2b-anti MSLN CAR-T cells exhibited enhanced migration and infiltration into tumor tissue, displaying superior anti-tumor efficacy, particularly in non-small-cell lung carcinoma [69]. In recent years, novel cytokine and chemokine receptors have been investigated in preclinical studies to improve CAR-T cell infiltration into tumors. For instance, CLDN18.2-specific IL-7 and CCL21 co-expressing CLDN18.2-specific cytokines (7 × 21 CAR-T cells) enhanced proliferation and chemotaxis of CAR-T cells across various cancer models such as PANC02 (pancreatic cancer), E0771-A2 (breast cancer), and Hepa1-6-A2 (liver cancer) [70]. Additionally, IL-7 and CCL19 expressing anti-GM2 CAR-T cells exhibited abundant CAR-T cell infiltration and strong therapeutic effects in GM2-positive solid cancers in xenograft models [71]. Trinh et al. designed a CX3CR1 overexpressing (NKG2D) CAR-T expression and found that CAR-T cells infiltrated tumors at higher rates than control-activated T cells or IL-15-overexpressing NKG2D CAR-T cells in a liver cancer model [72].

In clinical settings, regional delivery of CAR-T cells into tumors at various anatomical sites, including the brain [32], breast [73], pleura, and liver [74] has emerged as an alternative delivery strategy to enhance CAR-T cell localization in tumors. Prapa et al. compared intracerebral versus intravenous anti-GD2 CAR-T injections to treat glioblastoma, revealing that the intracerebral route significantly increased survival time in a dose-dependent manner without side effects [75]. Clinical data from the Phase 1b HITM-SIR trial demonstrated the safety and efficacy of CAR-T cell hepatic artery infusions therapy, which was not associated with severe cytokine release syndrome (CRS) or neurotoxicity [76, 77].

However, solid TMEs present physical barriers that CAR-T cells must overcome to penetrate tumors. One such barrier is the protease fibroblast activation protein (FAP), expressed by many tumor-associated stromal fibroblasts, which plays a crucial role in remodeling the tumor ECM. Targeting FAP-expressing stromal cells with CAR-T cells has been explored to facilitate immune cell infiltration into tumors. Nevertheless, results from studies are contradictory. While some studies reported limited effects on tumor progression and adverse effects on bone marrow stromal cells [78], others demonstrated decreased tumor growth without severe toxicities when combined with vaccines [79].

Apart from FAP targeting, engineering CAR-T cells to secrete ECM-modifying enzymes is another approach to enhance CAR-T cell penetration into solid tumors. For instance, anti-GD2 CAR-T cells engineered to degrade heparin sulfate proteoglycans in the ECM through heparinase expression exhibited improved tumor infiltration and prolonged survival compared to CAR-T cells lacking heparinase expression [80]. Other ECM-degrading enzymes are also under exploration for their potential role in CAR-T cell therapy. Recently, Wang et al. constructed a recombinant oncolytic vaccinia virus encoding hyaluronidase to degrade hyaluronic acid, which often impedes intratumoral dissemination of anti-tumor drugs. Their findings demonstrated increased intratumoral dissemination of chemo drugs, immune cell infiltration, and activation of CD8^+^ T cells [81]. While ECM modification presents an exciting frontier in CAR-T cell therapy for solid tumors, caution is warranted due to the complicated and currently unpredictable effects of ECM-modifying enzymes. Clinical trials have shown mixed results, indicating the need for further investigation into the efficacy and toxicity profile of these approaches. Additionally, there are concerns regarding potential thromboembolic events associated with ECM modification, as evidenced by the administration of low molecular weight heparin supplementation to mitigate risks in clinical trials [82, 83].

In summary, while significant strides have been made in enhancing CAR-T cell trafficking and infiltration into solid tumors, ongoing research is essential to further understand the complexities of the TME and optimize therapeutic strategies for improved efficacy and safety in clinical settings.

#### Blocking CAR-T cell dysfunction

Once located in the tumor, CAR-T cells must contend with direct inhibitory signals present in the TME. Multiple inhibitory signals, including the expression of PD-1 and other inhibitory receptors, have been identified as mechanisms of CAR-T cell dysfunction [84]. Interfering with checkpoint signal pathways is a common approach to alleviate CAR-T cell dysfunction and restore their anti-tumor efficacy [85, 86] (Fig. 2). A well-known inhibitory pathway is the PD-1–PD-L1 axis. PD-1 is an immune checkpoint receptor expressed in T cells. When PD-1 is bound by its ligand PD-L1 expressed on tumor cells as well as other cell types, it induces T cells to adopt an exhausted phenotype [87]. Combinational therapy of PD-1 antibody checkpoint blockade and CAR-T cells showed increased efficacy of CAR-T cell therapy in both preclinical and clinical settings [88]. Various groups have demonstrated increased efficacy of CAR-T cell therapy with the coadministration of antibodies that inhibit the PD-1 pathway in preclinical models [89, 90]. Jaspers et al. found that anti-PD-1 blockade increased memory phenotype, reduced exhaustion, and induced durable responses of anti-DLL3 CAR-T cells in multiple SCLC models [91]. Similarly, a Phase I clinical showed that anti-mesothelin CAR-T cells showed therapeutic effects in patients with malignant pleural disease, in combination with the anti-PD-1 agent pembrolizumab [92]. Instead of using CAR-T cells in combination with established ICIs, researchers also employed genetic engineering strategies to disrupt checkpoint pathways. Wang et al. generated PD-1 and TCR deficient mesothelin-specific CAR-T (MPTK-CAR-T) cells using clustered regularly interspaced short palindromic repeats (CRISPR) technology and evaluated them in a dose-escalation clinical study [93]. No dose-limiting toxicity or unexpected adverse events were observed in any of the 15 patients. The best overall response was stable disease (2/15 patients) [93]. Agarwal et al. found that CRISPR-mediated deletion of *CTLA4* permitted unopposed CD28 signaling and maintenance of CAR expression on the T cell surface under the condition of high antigen load [94]. CTLA4 deficiency in CAR-T cells improved proliferation and anti-tumor efficacy in preclinical models of leukemia and myeloma, which rescued the function of T cells from patients with leukemia that previously failed CAR-T cell treatment [94]. 4-1BB-based CAR-T cells armed with autocrine PD-L1 scFv antibody effectively reversed exhaustion and enhanced the anti-tumor immune response in solid tumors and hematologic malignancies by blocking the PD-1/PD-L1 signaling [95]. A similar study showed that mesothelin-targeting CAR-T cells secreting single-chain trimeric 4-1BB ligand fused to anti-PD-1 scFv (αPD1-41BBL) exhibited reduced inhibitory receptor upregulation; enhanced persistence, proliferation, and memory status; and augmented anti-solid tumor efficacy [96].

In the TME, various immuno-suppressive molecules, such as adenosine and TGF-β, produced by immuno-suppressive immune cells like Tregs and MDSCs, impose inhibitory roles on CAR-T cells [97]. CAR-T cells have been engineered to counteract the actions of adenosine, with CRISPR-mediated deletion of the adenosine A2A receptor enhancing CAR-T cell efficacy in several studies [65, 98]. Similarly, reducing adenosine levels through overexpression of adenosine deaminase has induced stemness and enhanced CAR-T functionality [99]. TGF-β inhibition via CRISPR or small molecule blockers such as LY2157299 has been shown to promote the long-term efficacy of CAR-T cells against solid tumors [93, 100]. Inactivation of TGF-β signaling in CAR-T cells through dominant-negative TGF-β receptor II has achieved optimistic preclinical and clinical results against solid tumors [101].

Another effective method to modify inhibitory signals in CAR-T cells is to express decoy or switch cytokine receptors, which converts inhibitory signals present in the TME into pro-inflammatory signals. For instance, switch receptors with chimeric signaling domains can convert TGF-β signals or PD-1/PD-L1 inhibitor signals through the engagement of modified receptors to signal through co-stimulatory domains such as 4-1BB or IL-12 [102, 103]. Similarly, cytokine receptors containing the extracellular domain of the IL-4 receptor fused with the endodomain of the IL-7 receptor can translate inhibitory IL-4 signals into activating IL-7 signals in T cells and CAR-T cells [104]. Another approach involves fusing the IL-4 receptor extracellular domain with the shared β-subunit of the IL-2 and IL-15 receptors, thereby converting inhibitory IL-4 signals into homeostatic IL-7, IL-2, or IL-15 signals [105]. These strategies represent innovative approaches to mitigate inhibitory signals present in the TME, thereby enhancing CAR-T cell efficacy in combating solid tumors.

#### Enhancing CAR-T cell anti-tumor function with immunostimulatory signals

An alternative strategy to enable CAR-T cells to overcome inhibitory signals in the TME involves engineering CAR-T cells with immunostimulatory signals to achieve enhanced anti-tumor functions (Fig. 2). The limited success of early iteration CAR-T cell designs lacking co-stimulation underscores the importance of incorporating a co-stimulatory domain. Currently, all CAR-T cells in clinical use contain either a CD28 or 4-1BB co-stimulatory domain [27]. Preclinical investigations are exploring the utility of including additional co-stimulatory molecules such as ICOS, OX40, and CD27, or various combinations of multiple co-stimulatory domains in third- and fourth-generation CAR constructs [106–109]. ICOS and 4-1BB co-stimulation have been shown to dramatically enhance CAR-T cell persistence [110]. Moreno-Cortes et al. generated ICOS.OX40 tandem co-stimulated anti-ROR1 CAR-T cells, demonstrating enhanced in vitro and in vivo cytotoxicity and prolonged persistence [111].

Augmenting CAR-T cells to secrete stimulatory cytokines represents another approach to enhancing CAR-T cell function. These “armored” CAR-T cells redirected for universal cytokine-mediated killing (TRUCKS) encode not only a CAR but also a cytokine, interleukin, pro-inflammatory ligand, or chemokine to counteract the immuno-suppressive microenvironment prevalent in most solid tumors [112–115]. Cytokines such as IL-12, IL-18, or IL-15 are commonly used to empower CAR-T cells with better in vivo proliferation, survival, and persistence. For example, IL-12 signaling facilitates CAR-T cell-mediated IFNγ production, which is necessary for tumor cell killing, and intratumoral IL-12 delivery enhances CAR-T cell immunotherapy in preclinical models of glioblastoma [116]. Lee et al. engineered membrane-bound IL-12 (mbIL12) in CAR-T cells, leading to increased proliferative capacity, better survival, and greater cytotoxicity compared to unarmored CAR-T cells, promoting durable anti-tumor responses in mice [117]. IL-18 augmented IFN-gamma secretion and proliferation of T cells activated by the endogenous TCR, enhancing anti-tumor activity [118]. Furthermore, IL-15 plays a significant role in T cell proliferation and persistence [45, 119, 120]. CAR-T cells armored with several other cytokines, such as IL-10 or IL-4/IL-15-based inverted cytokine receptors, have also shown significantly enhanced efficacy against solid tumors in preclinical models [121, 122].

#### Enhancing CAR-T cell function with endogenous genetic regulators

Moreover, with the advancement of CRISPR screens and single-cell sequencing technologies, various previously unknown or understudied T cell genetic regulators, such as suppressors and boosters, have been discovered recently [123, 124]. Genetic deletion of T cell negative regulators or overexpression of T cell boosters are powerful strategies to enhance the fundamental properties of T cells and thereby CAR-T cell anti-tumor functions. For instance, knockout of *Regnase-1* promotes TCF-1 expression to enhance CAR-T cell expansion and memory-like cell formation, reduce exhaustion, and support long-term CAR-T cell persistence [125]. Dong et al. discovered that RNA helicase *DHX37* knockout enhanced the efficacy of CD8^+^ T cells against tumors by regulating NF-κB [126]. *RASA2* ablation enhanced MAPK signaling and prolonged survival and cytolytic activity of CAR-T cells in mice xenografted with tumors [127]. Ye et al. identified proline metabolism (PRODH2) as a means to enhance CAR-T therapy through a genome-scale gain-of-function CRISPR screen in CD8 T cells [128]. A genome-scale open reading frame screen identified lymphotoxin-β receptor (LTBR) as a synthetic driver of T cell proliferation [129]. These regulators have shown promising efficacy in improving T cell fitness and anti-tumor efficacy and are generally universal across different types of CARs because they are fundamental genes in T cells [125–129]. Their therapeutic values await clinical testing.

In summary, engineering CAR-T cells with enhanced anti-tumor functions through co-stimulatory domains, cytokine secretion, and modulation of T cell regulators presents a promising avenue for improving CAR-T therapy against solid tumors. Continued preclinical investigations and future clinical trials will be crucial for translating these approaches into effective clinical treatments.

### Improving the clinical safety of CAR-T cells

Despite the clinical success of CAR-T therapy in hematological malignancies and the perspective to become a turning point also in solid tumors, this therapy still remains burdened by life-threatening side effects, such as CRS and neurotoxicity [130]. CRS is a systemic condition manifesting with constitutional symptoms, fever, hypotension, and organ dysfunction in the most severe cases [131]. It is provoked by a mass release of cytokines, with a crucial pathogenic role played by IL-1 and IL-6 [132], by CAR-T cells and TME cells upon cancer cell recognition by CAR-T cells [133]. CAR-T neurotoxicity, also known as immune effector cell-associated neurotoxicity syndrome (ICANS), is a toxic encephalopathy clinically closely linked to CRS in terms of pathogenesis [130]. The prevalence of CRS in the registration trials resulted as extremely high, inducing the conclusion that to a certain extent, all patients treated with CAR-T develop a CRS, while around half of the patients develop neurotoxicity [134]. It is worth mentioning that common experimental designs used to study in vivo CAR technology fail to adequately recapitulate this risk. This is demonstrated by the fact that CRS was first described in the clinical setting, without any anticipation by the preclinical studies [135–137]. While the assessment of CRS in mice requires the collection of straightforward parameters, including body temperature, weight, serum cytokines [132], and serum amyloid A3 [138], the detection of ICANS needs a more complex experimental evaluation, for which a consensus has not yet been achieved. In a milestone paper in which the crucial role of IL-1 and IL-6 in CRS and ICANS was highlighted, Norelli et al. evaluated histopathological signs of meningeal inflammation and defined lethal neurotoxicity as death preceded by motor deficit or seizures, in the absence of CRS criteria [132]. A more clinically detailed definition was provided by Faulhaber et al. who subjected the mice to a daily neurophenotype scoring and an open-field test, highlighting a correlation between ICANS and brain capillary obstruction [139]. Furthermore, a recent paper by Vinnakota et al., using a fully murine CAR-T cell model, described the activation of microglia after CAR-T injection. The described neuroinflammation can be reduced by interfering with the TGFβ-activated kinase1-NFκB-p38 MAPK pathway, ameliorating the neurocognitive deficits [140].

The current therapy for ICANS consists of a high-dose steroid course [141], which at the same time reduces the clinical effectiveness of CAR-T cells [142]. Therefore, an in-depth understanding of the pathophysiology of this manifestation would be highly beneficial to propose targeted therapy able to spare the CAR-T anti-cancer activity.

Another active study field is aimed at reducing the risk of off-target toxic activity, which could prove crucial for application in solid tumors. Among these, synNotch-regulated CAR enables T cells to express the CAR only in the presence of a specific priming antigen, increasing the specificity of the response while simultaneously preserving CAR-T cells from the tonic signaling provided by a constantly expressed CAR [143]. Similarly, by splitting the CD3ζ and co-stimulatory domains into CARs with different specificities, T cells can be engineered to be activated only in the concomitant presence of the two targets [144]. At the same time, also inhibitory CARs can prevent CAR-T cells from off-target activation [145]. The combination of both split activatory signaling (AND) and inhibitory CARs (NOT) allows for the integration of Boolean logic gates into cancer immunotherapy, enhancing the precision of targeting cancer cells.

Worth mentioning is a synthetic biology solution that can mitigate both on-target (CRS and ICANS) and off-target toxicity, consisting of switchable CAR-T cells that can be modulated to transition from inactive (OFF) to active (ON) state, or vice versa, through the administration of regulator molecules. These constructs can be grossly divided into two families, the OFF-switcher [146–148] and the ON-switcher [149–152]. The latter offers the additional advantage of protecting CAR-T cells from exhaustion induced by tonic activation [151].

## CAR-NK cell therapy

NK cells are innate lymphoid cells that play a critical role against tumors and viral infection [153]. NK cells recognize stressed cells [154] and exert a rapid and robust cytotoxic activity together with the production of inflammatory cytokines [155]. The activation of NK cells is regulated by a sophisticated integration of multiple germline-encoded receptors that provide either “kill” or “do not kill” signals [156]. The equilibrium between these opposing signals dictates NK cell responsiveness and is finely tuned during an education process aimed at achieving functional maturation and self-tolerance [157]. NK cells detect MHC class I (MHC-I) molecules through a variety of MHC-I-specific inhibitory receptors, including inhibitory killer cell immunoglobulin-like receptors (KIRs). The absence or low expression of MHC-I on the cell surface, which is frequently observed in tumor cells, can trigger NK-induced killing, known as “missing self” recognition [158]. Moreover, NK cells recognize ligands that are upregulated on the cell surface of stressed cells, known as “induced self” recognition. Besides “missing self” and “induced self” responses, antibody-dependent cellular cytotoxicity (ADCC) is another critical mechanism of NK-mediated recognition and killing of cancer cells. NK cells can mediate IgG opsonized cytotoxicity through CD16 (FCγRIII), which binds to the Fc portion of IgG antibodies [159] and induces cytotoxicity [160].

Upon activation, NK cells execute multiple functions to eliminate or constrain the growth of cancer cells. Interestingly, for their first killing events, NK cells eliminate target cells by forming lytic immunological synapses and secrete pre-assembled cytolytic granules containing granzyme B and perforin, leading to the apoptosis of the target cells [161, 162]. Later, NK cells switch to the use of death receptors, such as Fas ligand and TNF-related apoptosis-inducing ligand (TRAIL), to induce target cell death [163]. Moreover, NK cells can produce several pro-inflammatory cytokines, including IFN-γ and TNF-α, to limit the growth of target cells and orchestrate the function of other innate and adaptive immune cells [164].

NK cells were proposed as cellular immunotherapy for cancer over 30 years ago due to their diverse mechanisms for recognizing and eliminating cancer cells [165]. Furthermore, since NK cells do not recognize targets presented by the HLA system, NK cells-based immunotherapy can be used in an allogenic setting without the risk of graft-versus-host disease (GvHD) [166] making possible the development of a universal, “off-the-shelf” immunotherapy. These promising characteristics have recently sparked significant interest in CAR-NK cells, leading to exponential growth in technological advances and preclinical results. Leveraging decades of experience in T cell engineering, CAR-NK cells have undergone a significantly accelerated development toward clinical applications [167].

### Challenges in CAR-NK cell therapy for solid tumor

While the current clinical experience with CAR-NK cells mostly comes from hematological malignancies (as summarized in Table 1), insights into the biological characteristics of NK cells and the preclinical findings can help predict potential pitfalls in their application against solid tumors. Similar to CAR-T cells, the application of CAR-NK cells in solid tumors faces challenges related to trafficking toward the tumor. Despite playing a crucial role in orchestrating the anti-tumor responses [168], NK cells are often limited in their presence within several solid tumors [169], indicating difficulties in their ability to reach, infiltrate, and persist within TME. Additionally, infiltrating NK cells in solid tumors often display a dysfunctional phenotype [170–174], partly due to the detrimental factors such as hypoxia [175] and soluble inhibitory factors [176–178] present in the TME. Finally, NK cell-based immunotherapy must contend with the short half-life [179] and short-term anti-cancer activity [180]. Although the persistence of CAR-NK cells is a challenge shared with their application in hematological malignancies, it becomes even more crucial in the setting of solid tumors.

**Table 1:** Representative clinical trials evaluating CAR-NK cell therapy

Clinical trials evaluating CAR-NK cells						
Trial	NK source	CAR construct	Target	Disease	Outcome	Safety
NCT02944162 [236] (*n* = 3)	NK-92	CD28-4-1BB-CD3ζ	CD33	AML	No sustained responses	• 2 CRS (Grade 1) • No GvHD • No neurotoxicity
NCT03415100 [184] (*n* = 3)	Peripheral blood	NKG2D.CD8.DAP12	NKG2D-ligands	Colorectal cancer metastasis	Local response observed in all of the three patients (two patients with peritoneal carcinomatosis display ascites reduction and decrease in cancer cell number, the patient with liver metastasis achieved metabolic CR)	• 1 CRS (Grade 1) • No neurotoxicity • No GvHD • No serious adverse events (Grade ≥ 3)
NCT04245722 [300] (*n* = 20)	iPSC	NKG2D-2B4-CD3ζ-IL15RF-hnCD16	CD19	R/R B-cell tumors (B-NHL/CLL)	17 efficacy-evaluable patients Objective response = 9/17	• 2 CRS (Grades 1 and 2) • No neurotoxicity • No GvHD • No dose-limiting-toxicity
NCT04023071 [288] (*n* = 13)	iPSC	(Not entirely reported) hnCD16	CD20	R/R B-cell tumors (B-NHL/CLL)	Objective response = 8/13 CR = 7/13	• No CRS • No neurotoxicity • No GvHD • No CAR-NK cells-related Grade ≥ 3 adverse events • No dose-limiting-toxicity
NCT03056339 Dose-escalation phase [216] (*n* = 11) Expansion phase [217] (*n* = 26)	Cord blood	CD28-CD3ζ.iCasp9-IL-15	CD19	R/R B-cell tumors (B-NHL/CLL; ALL; LL)	Day 100 OR = 48.6% 1-year-OS = 68%	• 1 CRS (Grade 1) • No neurotoxicity • No GvHD • Reversible Grades 3 or 4 hematological toxicity (likely induced by the lymphodepleting chemotherapy)

*hnCD16a* high-affinity non-cleavable variant of CD16a, *NHL* non-Hodgkin lymphoma, *CLL* chronic lymphocytic leukemia, *AML* acute myeloid leukemia, *ALL* acute lymphoblastic leukemia, *LL* lymphoplasmacytic lymphoma, *OR* overall response, *OS* overall survival

### Strategies to improve CAR-NK cell therapy for solid tumors (Fig. 3)

#### Increasing NK trafficking and infiltration to solid tumors

Clinical evidence suggests that the presence of activated NK cells infiltrating solid tumors correlates with positive prognostic outcomes [181–183], despite as aforementioned their low ability to infiltrate and persist [169]. For this reason, in the first clinical study evaluating CAR-NK cells in solid tumors, the cells were locally administered in the colon cancer metastatic sites [184]. The three patients treated showed signs of local response, including a drastic reduction of cells in the ascites and a complete metabolic response of the liver metastasis. However, since the higher risk of adverse events and the logistic difficulties related to locoregional delivery compared to intravenous injection, various strategies have been tested to enhance NK cell migration.

**Fig. 3:**
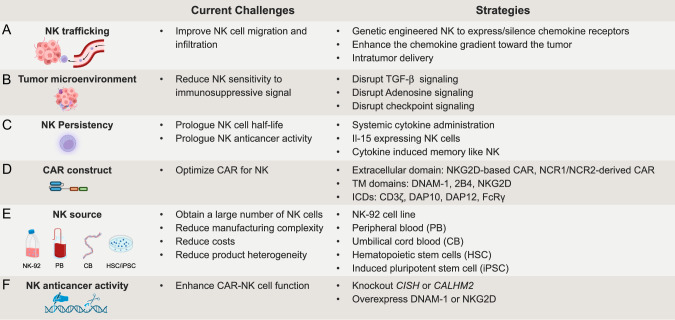
Current challenges and ongoing strategies to improve CAR-NK cell therapy for solid tumors. CAR-NK cells face common obstacles with CAR-T in effectively targeting solid tumors, including **A** cell trafficking and **B** the immuno-suppressive effects of the tumor microenvironment Potential strategies to overcome these shared challenges are closely linked to understanding NK biology in the context of solid tumors. NK cells are known for their short half-life and time-limited anti-cancer activity (**C**), and overcoming these biological characteristics may represent a turning point for the application to solid tumors. Indeed, the reduced availability of direct contact with cancer cells in solid tumors compared to hematological ones makes the generation of long-surviving and long-acting NK cells crucial. CAR-NK cells have benefited from the translation of well-consolidated designs optimized for CAR-T. In recent years, increased attention has been focused on different strategies to optimize CAR specifically for NK cells (**D**). Transmembrane (TM) and intracellular co-stimulatory domains (ICDs). **E** Different sources have been explored to obtain NK cells for clinical applications, including the immortalized NK-92 line, peripheral blood (PB), cord blood (CB), hematopoietic stem cells (HSC), and induced pluripotent stem cells (iPSC). Each of these sources encompasses advantages and limitations. The challenge of obtaining CAR-NK cells with the optimal balance between pharmacoeconomic sustainability (cost and manufacturing complexity) and reproducible efficacy (homogeneous and effective product) is a critical step to fully harness their potential as an off-the-shelf therapy. **F** Gene editing strategies aimed at boosting NK cell anti-cancer activity have begun to be explored in both hematological [249] and solid tumor models [250, 251]. Based on the swift advances observed in this field for CAR-T cells, it is likely that the biotechnological solutions to boost NK cell activity will exponentially increase in the coming years

Genetic engineering CAR-NK cells to express the chemokine receptor CXCR4 has resulted in increased migration toward CXCL12/SDF-1α and enhanced in vivo efficacy against CXCL12/SDF-1α-secreting glioblastoma model U-87 [185]. Overexpression of CXCR1 in CAR-NK cells has shown increased migration toward the pro-inflammatory cytokine IL-8 gradient, resulting in increased infiltration in peritoneal ovarian cancer xenografts [186]. Furthermore, CXCR2-transduced NK cells have demonstrated increased migration along CXCR2 ligands or renal cell carcinoma tumor supernatants [185]. Similar results can be achieved by inhibiting the G protein-coupled receptor CXCR3, involved in the mobilization of NK cells from the bone marrow to the PB, thereby increasing NK infiltration in a multiple myeloma model [187]. An alternative strategy proposed by Lee et al. is the NK cell-recruiting protein-conjugated antibody [188]. This molecule releases CXCL16 once cleaved by furin expressed on the surface of pancreatic cancer cells, creating a gradient that enhances NK migration toward the tumor in vivo.

#### Overcoming TME immunosuppression

Unlike dispersed hematologic malignancies, solid tumors, especially late-stage ones, exhibit an inhibitory TME due to hypoxia, a low pH, presence of suppressive cytokines, lactate, prostaglandins, and others that negatively impact NK cell function [189]. TGF-β dampens various aspects of NK cell anti-tumor function, including cytokine secretion, degranulation, metabolism, and mTOR signaling [190–192]. Therefore, pharmacological inhibition of TGF-β [193] or engineering the TGF-β receptor on NK cells can potentially overcome TGF-β-mediated inhibition and enhance NK anti-cancer activity [194–196]. With this rationale, Prof. Weathers’s group started a Phase I clinical trial evaluating the use of NK cells genetically depleted for the TGF-β receptor 2 and the endogenous glucocorticoid receptor NR3C1 (ClinicalTrials.gov Identifier: NCT04991870). If this trial shows positive results, this strategy can also be implemented in CAR-NK to improve the detrimental effect of TME.

NK anti-tumor function is also affected by extracellular adenosine, an element of hypoxia-driven purinergic signaling [197]. Adenosine is generated in high concentrations in the TME from ATP and ADP by the sequential activity of CD39/CD73 ectonucleotidases, providing a broad immuno-suppressive effect on both T cells and NK cells [198, 199]. Specifically, CD39-expressing Tregs are capable of inhibiting the anti-tumor immunity of NK cells both in vivo and in vitro [200]. Different strategies can be pursued to prevent the detrimental role of adenosine on NK cells, including ectonucleotidase or A2 receptor inhibitors [201]. Furthermore, lymphocytes can be engineered to suppress the expression of adenosine receptors [202–204].

As described for CAR-T, CAR-NK can also benefit from the concomitant use of checkpoint inhibitors. Both CTLA4 and PD-1 are expressed by activated NK cells [205, 206] and NK anti-cancer activity can be enhanced by disrupting these signaling pathways [207, 208]. Furthermore, additional checkpoint inhibitors, including NKG2A, TIGIT, and TIM3, have been shown to negatively impact NK function [209, 210] and conversely, their inhibition may improve NK anti-cancer activity [211–213].

#### Increasing NK persistency

One of the primary challenges in the clinical application of NK cells for immunotherapy is their short half-life post-infusion, typically limited to a few weeks [214, 215]. Currently, the most successful strategy in the clinical setting involves NK cells encoding IL-15, which have demonstrated in clinical settings, the persistence for over 1 year after infusion [216]. These data were reported in the dose-escalation phase of the trial evaluating CAR-NK cells in B-cell tumors, enforced by the observation that responder patients display a longer persistence of the injected cells [217].

Once these data were integrated with the expansion phase (37 patients in total), the CD19/IL-15 CAR-NK demonstrated an overall response rate of 48.6 and 68% of the patients survived for at least 1 year. This indicates an effectiveness comparable to the current CAR-T cell therapies [27], but notably no major toxicity events such as severe CRS, neurotoxicity, or GvHD were observed [217].

Furthermore, the Rezvani group has also provided mechanistic insights by integrating the post-infusion transcriptomic profile obtained from the clinical trial with a preclinical model of non-curative lymphoma [218]. Armoring CAR-NK with IL-15 appears to ameliorate the loss of metabolic fitness observed after cell infusion, enhancing in this way the tumor control in vivo.

The inclusion of IL-15 into the CAR construct represents a promising strategy also in other hematological malignancies [219], and solid tumors, where these cells have shown a long persistence in vivo in a preclinical model of pancreatic cancer [220].

Another promising option for allogeneic cell therapy is harnessing memory NK cells. This concept arises from evidence suggesting that NK cells exhibit features of adaptive memory under specific circumstances [221]. Like memory B and T cells, memory NK cells possess the ability to mount an enhanced response upon rechallenge to the same stimulus encountered previously and can be maintained by a long-lived/self-maintaining population [222]. Cytokine-induced memory-like (CIML) NK cells are generated through ex vivo priming with IL-12, IL-15, and IL-18. These cells exhibit prolonged persistence and demonstrate higher anti-cancer activity after a resting period, encouraging investigation in clinical settings. Indeed, CIML NK cells have shown encouraging results in clinical settings [223–225] and represent a promising strategy as a platform for CAR-NK technology [226].

#### Optimizing CAR constructs for NK signaling (Fig. 4)

Currently, most CAR-NK cell studies and clinical trials simply adapt CAR constructs that were originally designed for CAR-T cells. While these constructs can also function in NK cells, they are not optimized for NK signaling. Given the variety of activating receptors and adapter protein domains that contribute to NK fine-tuned activation, NK CAR constructs could have more variations and different combinations in the choice of extracellular, TM, and intracellular signaling domains. This approach could lead to the development of more effective and tailored CAR-NK cells for cancer immunotherapy.

**Fig. 4:**
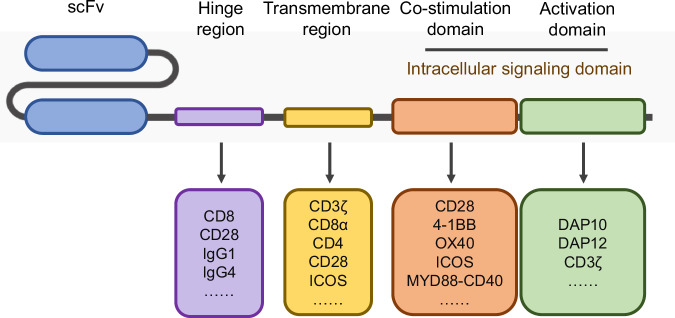
Blueprint of CAR-NK construct design and optimization. Like CAR-T, CAR-NK cells follow a modular design comprising an antigen-binding domain, a hinge, a transmembrane domain, and an intracellular signaling domain. Design and optimization strategies can also be specific to each domain. In the hinge region, options such as CD8, CD28, IgG1, and IgG4 have demonstrated some efficacy in CAR-T cells and merit consideration. Additionally, for the transmembrane domains, while CD3ζ, CD8α, CD4, CD28, and ICOS are commonly employed in CAR-T cells, receptors specific to NK activation, such as CD16, NKp44, NKp46, NKG2D, 2B4, and DNAM-1, should be explored. When it comes to co-stimulation domains, candidates like CD28, 4-1BB, OX40, ICOS, and MYD88-CD40 warrant investigation. Furthermore, for the activation domain of the intracellular signaling module, besides CD3ζ, NK-specific activating receptors, DAP10, and DAP12, offer promising avenues for experimentation

##### Extracellular scFv domains

Single and tumor-specific targets are challenging to find due to the expression of most so-called TAAs on solid tumors are often expressed at lower levels in healthy tissue. Moreover, targeting a single antigen might lead to immune escape, as often observed in CAR-T therapy. Notably, NK-activating receptors naturally have less specificity and can target multiple cancer antigens. NKG2D, for example, can recognize up to eight stress-induced molecules, including MICA/B and RAET1/ULBP family, on the surface of distressed cells [227]. Therefore, certain CAR constructs exploit the natural tumor recognition of NK receptors. NKG2D-based CAR-NK cells have been shown to be effective against multiple myeloma cells in a preclinical setting [228] and in a clinical study with metastatic colorectal cancer patients [184]. Similarly, NCR1-derived CAR or NCR2-derived CAR constructs are tested to improve cancer immunotherapy [229, 230]. These approaches leverage the broader tumor recognition capabilities of NK cells to potentially overcome the limitations associated with targeting single antigens, benefiting both CAR-T and CAR-NK cell therapy.

##### Transmembrane domains and hinge

The TM domain serves to connect the extracellular domain of the CAR to the intracellular activation signaling domains and dock the CAR construct to the cell membrane. Therefore, they are important for signal transduction CAR dimerization and signal transduction. In CAR-NK cells, the most commonly used TM domains are adapted from CAR-T cell constructs, such as CD3ζ, CD8, and CD28. However, the TM domains of several NK-activating receptors, such as CD16, NKp44, NKp46, NKG2D, 2B4, and DNAM-1, possess charged amino acids capable of directly interacting with signaling adapters and follow the natural orientation (N to C terminal, except for NKG2D). Consequently, these TM domains are actively explored in the NK CAR screen studies [231]. Interestingly, CAR constructs containing TM domains from NK-activating receptors, such as DNAM-1, 2B4, and NKG2D, typically yield higher cytotoxicity.

##### Intracellular co-stimulatory domains

Once the signals from activating receptors surpass those provided by inhibitory receptors [232], NK cells are activated through signaling adapter proteins. These proteins, in addition to CD3ζ, include DAP10, DAP12, and FcRγ [233]. The incorporation of these activated signaling adapters, either individually or in combination, to optimize CAR design for NK cells has begun to be explored in recent years [234]. Li et al. identified a tailored NK CAR construct containing the NKG2D TM domain, the 2B4 co-stimulatory domain, and the CD3ζ signaling domain, which mediated stronger antigen-specific cytotoxicity than T CAR and other constructs [231]. Moreover, CAR constructs including other NK-specific signaling domains, such as DAP10 and DAP12, could also increase NK anti-tumor efficacy [231]. These findings underscore the importance of optimizing CAR constructs specifically for NK cells to enhance their therapeutic potential. However, there is a lack of systematic research on NK CAR constructs, and the full potential of NK CAR-mediated signaling has yet to be fully explored.

#### Novel expansion methods and sources of NK cells

To date, various sources have been explored to obtain NK cells for clinical applications, including the immortalized cell line NK-92, umbilical cord blood (CB), CB-derived CD34^+^ hematopoietic stem and progenitor cells, and induced pluripotent stem cells (iPSCs). Each of them presents advantages and disadvantages in terms of the linked manufacturing processes, including source collection, NK isolation/differentiation, genetic modification, and expansion into large amounts.

##### Immortalized NK cell lines

NK-92 cells, derived from a patient with non-HL, offer the potential for unlimited ex vivo expansion with minimal manufacturing effort [235]. Moreover, due to their homogeneous nature and lower resistance to transduction, NK-92 cells serve as an ideal prototype for developing CAR-based immunotherapies [236]. Consequently, NK-92 cells were the first NK cell line to gain authorization from the FDA for clinical trials and the platform for the first-in-man trial involving CAR-NK [236]. An interim report of this Phase I clinical trial evaluating CD33-CAR NK-92 cells in three patients with relapsed or refractory acute myeloid leukemia reported no significant adverse event but also no durable disease control [236].

Indeed, due to their immortalized nature, NK-92 cells require irradiation before clinical use, resulting in a subsequent reduction in persistence and clinical efficacy [237]. Furthermore, NK-92 cells lack ADCC due to the absence of CD16 expression [238]. Therefore, the development of genetically engineered NK-92-derived products is essential for enhancing their in vivo anti-tumor function.

##### Peripheral blood (PB)-derived NK cells

Primary NK cells can be isolated and expanded from PB, where they commonly represent 5–15% of the lymphocytes [239]. To obtain the required amount of cells for clinical applications, this protocol requires the apheresis of a healthy donor and has been the main source of non-genetically modified NK cells in recent years, obtained through both negative selection after CD3/CD19 depletion or positive selection [240].

##### Umbilical cord blood (CB)-derived NK cells

Unlike PB, CB lymphocytes contain up to 30% NK cells and a higher percentage of CD56^bright^ subset, making CB a rich source of therapeutic effector NK cells [241]. Furthermore, CB is abundant in HPCs, making it an ideal substrate for the in vitro differentiation of therapeutic NK cells with desired phenotypes [242]. However, CB-tderived NK cells show weaker cytotoxic activity against K562 leukemia cells and produce less IFN-γ compared to PB-derived NK cells, possibly due to their higher expression of inhibitory receptors such as NKG2A [241, 242]. Further exploration is needed to understand the regulators guiding CB HPC differentiation into effector NK cells with enhanced in vivo anti-tumor efficacy. The therapeutic potential of CB-derived NK cells requires systematic evaluation in more clinical trials.

##### Hematopoietic stem cells (HSC)-derived NK cells

To date, most adoptive NK cell therapies have utilized PB NK cells, CB NK cells, or NK-92 cells. However, each of these cell sources presents significant limitations, as discussed earlier. Due to the issues of clinical efficacy and donor heterogeneity, considerable attention is now directed toward stem cell-derived NK cells, offering standardized “off-the-shelf” therapies for patients regardless of their HLA haplotype. NK cells expanded and differentiated from CD34^+^ HSC offer the potential for unlimited quantities and are more amenable to genetic manipulation [243]. Although this method inherits many logistical advantages from using CB as a source for NK expansion, it requires more than 40 days of expansion time due to the relatively low absolute number of CD34^+^ cells retrieved from CB [155]. Overall, these sources have undergone testing in Phase I clinical trials for acute myeloid leukemia, showing promising results [243].

##### Induced pluripotent stem cell (iPSC)-derived NK cells

The iPSC-based methodology involves reprogramming adult somatic cells into pluripotent cells, followed by differentiation into NK cells and subsequent expansion to generate the final products [244]. iPSCs can be derived from easily accessible cells such as fibroblasts and blood cells, offering the potential to generate a large number of homogeneous NK cells from a single clone. This approach addresses the variability associated with editing a bulk NK cell population, making iPSCs-derived NK particularly suitable for gene-edited products [245–247]. Additionally, iPSC-derived NK cells have been shown to exhibit more potent cytotoxicity than PB NK cells [231]. Currently, iPSC-derived NK cells with multiple genes modified are being actively evaluated in clinical trials [248].

#### Alternative strategies for enhancing CAR-NK cell function

To date, only a small number of genes have been identified where knockout or perturbation has a strong effect on NK cell’s anti-tumor efficacy. For example, the deletion of *Cytokine-inducible SH2-containing protein* (*CISH*) in iPSC or CB-derived NK cells enhances in vivo persistence and anti-tumor response [218, 249]. It has also been shown that overexpressing DNAM-1 and/or NKG2D in NK cells enhances effectiveness against patient-derived sarcoma specimens in vitro [250]. The complex interaction among various mechanisms can interfere with NK activity in the setting of solid tumors, posing challenges for reductionist approaches to exploring these issues. On the other hand, this complex scenario is particularly suitable for unbiased functional genetic screens using in vivo models that can recapitulate the interrogated phenotype. We have recently conducted a study in which an in vivo CRISPR screen and orthogonal single-cell profiling of infiltrating NK cells across different tumor models have convergently identified the novel NK suppressor *Calcium homeostasis modulator family member 2* (*CALHM2*). Knockdown of this calcium channel resulted in improving CAR-NK anti-cancer efficacy both in murine and human NK cells [251]. Finally, CAR-NK cells have been shown to acquire the target antigen on their surface through a trygocytosis process [252]. This event not only reduces the presence of the tumor antigen in the target cells but also induces a fratricide depletion of the therapeutic cells. To prevent this process and enhance cell activity, CAR-NK cells can be engineered to express a second inhibitory KIR-based CAR, able to recognize a “do not kill me” signal like the NK-expressed CS1 antigen [253]. This strategy has been demonstrated to improve in vivo the CAR-NK cell anti-cancer activity against B-cell lymphoma [252].

## CAR-T vs CAR-NK cells

### Advantages of CAR-NK cells over CAR-T cells (Fig. 5)

One of the major limitations of the broader applicability of CAR-T therapy is linked to the necessity to use donor-derived cells to avoid both GvHD and the rejection of the infused cells, known as host-versus-graft (HvG) reaction. Since patients are usually heavily treated before CAR-T therapy, harvesting sufficient healthy autologous T cells from patients can significantly delay treatment and pose challenges for CAR-T manufacturing. The lengthy and cumbersome process of CAR-T manufacturing makes many patients either ineligible for the treatment or experience disease progression after being enrolled in the treatment process [254]. It must be mentioned that for hematological tumors previously treated with allogeneic hematopoietic transplantation, CAR-T cells derived from the same donor can be used without evidence of GvHD [255]. However, the necessity to produce a patient-specific product deeply impacts the possibility of scaling up the manufacturing process to reduce the cost, which is estimated in the USA between $370,000 and $530,000 per single treatment [256]. Therefore, the possibility of using an “off-the-shelf” allogeneic product currently represents a priority in this field.

**Fig. 5:**
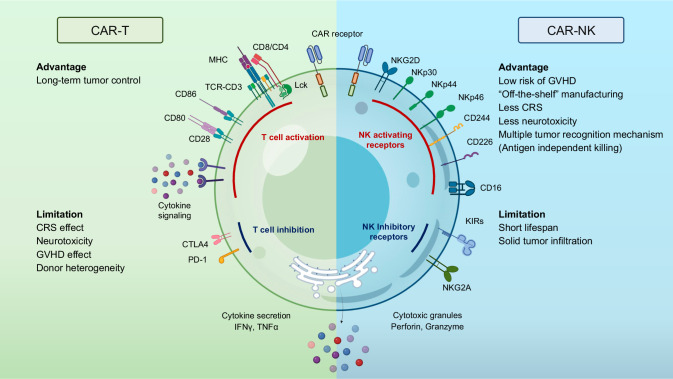
Advantages and limitations of CAR-T and CAR-NK cells. Both CAR-T and CAR-NK cells exhibit distinct advantages and limitations. CAR-T cells offer long-term tumor control capabilities but are associated with risks such as cytokine release syndrome, neurotoxicity, GvHD effects, and donor heterogeneity. On the other hand, CAR-NK cells present several advantages, including a lower risk of GvHD, “off-the-shelf” manufacturing feasibility, reduced incidence of cytokine release syndrome and neurotoxicity, as well as antigen-independent killing abilities. However, they do have drawbacks such as a shorter lifespan and potentially limited tumor infiltration capabilities

Different strategies can be pursued to reduce and limit the risk of CAR-T cells-induced GvHD. This adverse event mainly depends on the expression of functional αβTCR, therefore CAR-T cells can be genetically modified to not express the TCRα chain [257–260] and used as an allogeneic product. Since CAR-NK cells act in an HLA-unrestricted manner, these cells have the advantage of not inducing GvHD in an allogenic setting and therefore do not require any additional gene editing to be used as an “off-the-shelf” therapy. Existing NK-92 cell lines or primary NK cells sourced from allogeneic donors can be utilized and manufactured through mass production, as demonstrated by Rezvani’s group which has proposed a protocol able to obtain more than a hundred CAR-NK cell doses from one CB unit [261].

On the contrary, both allogenic TCRα chain^−^ CAR-T and CAR-NK cells remain vulnerable to HvG reaction when administered in immunocompetent patients. In hematological malignancies, the possibility of disrupting the recipient’s immune system as part of the therapeutic process has allowed different options to avoid rejection. Among these, knocking out the common lymphocyte antigen CD52 [262] on CAR cells and administering them together with the lymphodepleting anti-CD56 antibody alemtuzumab is a viable strategy [263]. However, in conditions like solid tumors, the necessity to preserve also the host immune system implies pursuing different approaches. Gene editing approaches aimed to make invisible these cells to the immune system must contend with the robustness of immunosurveillance. Indeed, while the knockout of β2-microglobulin can counteract the HLA-dependent recognition of recipient T cells [262], it provides a “missing self” activating signal to the NK cells. This issue could be avoided by overexpressing the non-classical HLA-E or G [262, 264] but this requires an additional gene editing step.

#### Safety profile with reduced CRS and neurotoxicity

CAR-T cell activation often leads to the release of inflammatory cytokines, resulting in CRS and neurotoxicity [254, 265]. However, CAR-NK cells release different profiles of cytokines. Indeed, as consistently demonstrated by a long series of clinical trials with parental NK cells, patients who received allogenic CAR-NK cell therapy did not develop CRS or neurotoxicity and did not exhibit increased levels of pro-inflammatory cytokines, such as IL-1β, IL-6, and IL-10 [214, 266–268]. Despite the current extremely limited clinical experience with CAR-NK compared to CAR-T cells [184, 217, 236], these clinical data so far confirm what has been observed for parental NK cells and have shown that, in contrast to CAR-T, CAR-NK cells have been rarely associated with CRS and not associated with neurotoxicity [217, 269]. This suggests that CAR-NK cells may offer a safer alternative to CAR-T cells about these serious adverse events.

#### Multiple tumor recognition mechanisms

Since CAR-NK cells can recognize and eliminate cancer cells in both antigen-dependent and antigen-independent manners [270] this therapy may present significant advantages in the application for solid tumors compared to CAR-T. As previously mentioned, different strategies are currently under evaluation to allow CAR-T to overcome the heterogeneity and antigen escape of solid tumors. In contrast, NK cells naturally possess antigen-independent mechanisms that may prove beneficial for tumors with fine subclonal heterogeneity [271]. Additionally, NK cells can mediate ADCC through CD16, which binds to the Fc domain of IgG bound on cancer cells. This further enhances their potential effectiveness against solid tumors.

### Limitations of CAR-NK cells

Despite CAR-NK’s promising features, several significant limitations exist that hinder its successful application in treating solid tumors. Many of these limitations parallel those associated with CAR-T therapy, such as poor tumor infiltration [272, 273] and detrimental interactions with the TME [189]. Moreover, NK cells have specific limitations unique to their biology and function.

#### Resistance to viral transduction

One of the main limitations of CAR-NK cell application consists of the resistance of NK cells to viral transduction [274] compared to T cells [275]. In a recent clinical trial using a retrovirus vector to generate CAR-NK cells a median transduction efficiency of 72.4% was observed [217]. However, the wide range of variability in these data (from 22.7% to 91.1%) represents a significant limitation that could affect the statistical power of a controlled Phase II or Phase III clinical trial. This result was obtained by stimulating NK cells with IL-2 and feeder cells the day before the transduction, taking advantage of the retrovirus’s preference for infecting actively replicating cells [276]. Among the six CAR-T cell products currently approved by FDA, four adopt a lentivirus vector (Carvykti™, Kymriah™, Breyanzi®, and Abecma®), and two a γ-retroviruses one (Yescarta™ and Tecartus™). Despite there are currently no clinical data indicating that one strategy is superior or safer than the other, at least for the current generation of viral vectors, it must be noted that γ-retroviruses preferentially insert near enhancers and promoters, implying a higher risk of insertional mutagenesis compared to lentivirus vectors [277]. This phenomenon dramatically emerged in clinical settings when children affected by X-linked severe combined immunodeficiency treated with γ-retrovirus-edited HSCs developed leukemia [278–280]. For this reason, different strategies have been explored to optimize also a lentivirus-based transduction protocol for NK cells. These strategies must address the antiviral response that lentivirus triggers in NK cells, which induces their apoptosis [281]. Among them, worthy of mention is the exposure to cytokine combinations like IL-2, IL-12, and IL-21 [281–283] or the pretreatment with the TBK1-inhibitor BX759, able to interfere with the signal downstream the pattern recognition receptors [281, 284]. The results from these studies have demonstrated the possibility of increasing lentivirus transduction efficiency in human primary NK cells to 25–40% [281, 284], therefore further research and innovative solutions are needed to optimize this process in NK cells.

#### Short lifespan and poor persistence

NK cells have a short half-life [285] and exhibit poor persistence once transferred from ex vivo conditions with supraphysiological cytokine exposure to in vivo conditions [286]. This aspect could potentially imply the necessity for multiple infusions. For CAR-T cells, a second infusion is not associated with major toxicity risks [287], but this strategy has only started to be explored for CAR-NK cells. Current evidence seems to indicate that multiple administrations of CAR-NK are not associated with serious adverse events. However, these data come from the interim reports of three Phase I clinical trials, performed in solid tumors [184] and hematological malignancies [236, 288], that together enrolled a total of 19 patients. Furthermore, it must be mentioned that in the first study, the cell product only transiently expressed the CAR, as it was obtained by electroporating NK cells with NKG2D CAR mRNA and was locally injected into the metastatic sites. Therefore, further studies are needed for the safety evaluation of this strategy. Furthermore, this approach would potentially impact the cost-effectiveness of the CAR therapy, therefore there is an active exploration into reprogramming CAR-NK cells to possess memory or memory-like properties, aiming for long-term tumor control. This avenue of research holds promise for addressing the limitations associated with NK cell persistence in CAR-NK cell therapy.

## Future perspectives

FDA has recently issued a warning on approved CAR-T cell products due to the observation of T cell malignancies in a small number of CAR-T-treated patients. Despite the overall low rate of such events (22 cases reported among over 27,000 treated patients reported to date), the recurrent reports across 5 of the 6 approved CAR-T cell therapy products have led the FDA to add a class-wide boxed warning to these therapies [289]. In three of these cases, cancer cells have been demonstrated to contain the CAR [290], indicating a malignant transformation of the infused therapy as the pivotal cause. This malignant transformation appears to be a rare event, however, since the current perspective of a broad expansion of clinical indication do CAR therapy, encompassing cancers in earlier therapeutic stages and other conditions, including autoimmune diseases [291], and HIV [292], the assessment of rare adverse events will become even more pertinent. At the moment we are writing this review, the mechanistic events leading to the cancerous transformation of CAR-T cells are under investigation, and no firm conclusions have been drawn on the direct involvement of the virus vector in this process [290]. However, the FDA warning represents an opportunity to review all the critical steps of CAR manufacturing, including vector safety, and evaluate potential solutions.

A proposed strategy to address these safety concerns includes the incorporation of a suicide gene, like caspase 9 [64] or the herpes simplex virus tyrosine kinase [293] to induce apoptosis in the event of toxicity or malignant transformation. Furthermore, alternative delivery systems are under investigation in clinical trials, including CRISPR/Cas9 and transposon/transposase-based approaches [294–297]. Both strategies present advantages and disadvantages, for which an in-depth discussion can be found in a dedicated review [298], however, it is evident that these technologies are living in an exponential development phase that will likely surpass the retro/lentivirus-based approaches in terms of safety, quality of the product (e.g., efficiency of transgene delivery), logistic feasibility and costs. For example, in our laboratory, we have developed a transposon/transposase-based system, named MAJESTIC (mRNA Adeno-associated virus-Sleeping Beauty Joint Engineering of Atable Therapeutic Immune Cells). By making as transient the Sleeping Beauty transposase component, this system avoids repeated transposon mobilization, further reducing the risk of insertional mutagenesis [299].

The contribution of innovative biotechnological tools to the improvement of CAR immunotherapies has only begun to show its potential. For example, many of the solutions to overcome the limitations of CAR-T and NK cells summarized in this review have been achieved through the application of high-throughput CRISPR technology [123, 124, 126, 128, 129, 251]. Furthermore, data derived from ongoing clinical trials will allow for a deeper understanding of the limitations of CAR therapies, enabling the design of more precise readouts for functional genetic screens. This dynamic and virtuous exchange between biotechnology development and data from clinical trials represents the keystone for evolving CAR-T and CAR-NK therapies toward the next generations of cancer treatment, ultimately leading to improved patient outcomes.

## Supplementary information

clinical trial search results

## References

[1] Riley RS, June CH, Langer R, Mitchell MJ. Delivery technologies for cancer immunotherapy. Nat Rev Drug Discov. 2019;18:175–96. 30622344 10.1038/s41573-018-0006-z PMC6410566

[2] Zhang Y, Zhang Z. The history and advances in cancer immunotherapy: understanding the characteristics of tumor-infiltrating immune cells and their therapeutic implications. Cell Mol Immunol. 2020;17:807–21. 32612154 10.1038/s41423-020-0488-6 PMC7395159

[3] Ma R, Li Z, Chiocca EA, Caligiuri MA, Yu J. The emerging field of oncolytic virus-based cancer immunotherapy. Trends Cancer. 2023;9:122–39. 36402738 10.1016/j.trecan.2022.10.003 PMC9877109

[4] Maalej KM, Merhi M, Inchakalody VP, Mestiri S, Alam M, Maccalli C, et al. CAR-cell therapy in the era of solid tumor treatment: current challenges and emerging therapeutic advances. Mol Cancer. 2023;22:20. 36717905 10.1186/s12943-023-01723-z PMC9885707

[5] Naimi A, Mohammed RN, Raji A, Chupradit S, Yumashev AV, Suksatan W, et al. Tumor immunotherapies by immune checkpoint inhibitors (ICIs); the pros and cons. Cell Commun Signal. 2022;20:44. 35392976 10.1186/s12964-022-00854-y PMC8991803

[6] Yoo MJ, Long B, Brady WJ, Holian A, Sudhir A, Gottlieb M. Immune checkpoint inhibitors: an emergency medicine focused review. Am J Emerg Med. 2021;50:335–44. 34450397 10.1016/j.ajem.2021.08.038

[7] Fan A, Wang B, Wang X, Nie Y, Fan D, Zhao X, et al. Immunotherapy in colorectal cancer: current achievements and future perspective. Int J Biol Sci. 2021;17:3837–49. 34671202 10.7150/ijbs.64077 PMC8495390

[8] O’Donnell JS, Teng MWL, Smyth MJ. Cancer immunoediting and resistance to T cell-based immunotherapy. Nat Rev Clin Oncol. 2019;16:151–67. 30523282 10.1038/s41571-018-0142-8

[9] Larson RC, Maus MV. Recent advances and discoveries in the mechanisms and functions of CAR T cells. Nat Rev Cancer. 2021;21:145–61. 33483715 10.1038/s41568-020-00323-z PMC8353572

[10] Shiravand Y, Khodadadi F, Kashani S, Hosseini-Fard SR, Hosseini S, Sadeghirad H, et al. Immune checkpoint inhibitors in cancer therapy. Curr Oncol. 2022;29:3044–60. 35621637 10.3390/curroncol29050247 PMC9139602

[11] Boukouris AE, Theochari M, Stefanou D, Papalambros A, Felekouras E, Gogas H, et al. Latest evidence on immune checkpoint inhibitors in metastatic colorectal cancer: a 2022 update. Crit Rev Oncol Hematol. 2022;173:103663. 35351582 10.1016/j.critrevonc.2022.103663

[12] Fuchs N, Zhang L, Calvo-Barreiro L, Kuncewicz K, Gabr M. Inhibitors of immune checkpoints: small molecule- and peptide-based approaches. J Pers Med. 2024;14:68. 38248769 10.3390/jpm14010068 PMC10817355

[13] Finck AV, Blanchard T, Roselle CP, Golinelli G, June CH. Engineered cellular immunotherapies in cancer and beyond. Nat Med. 2022;28:678–89. 35440724 10.1038/s41591-022-01765-8 PMC9305718

[14] Sun Q, Zhao X, Li S, Yang F, Wang H, Cui F, et al. CSF neurofilament light chain elevation predicts ALS severity and progression. Front Neurol. 2020;11:919. 32982935 10.3389/fneur.2020.00919 PMC7484044

[15] Schreiber RD, Old LJ, Smyth MJ. Cancer immunoediting: integrating immunity’s roles in cancer suppression and promotion. Science. 2011;331:1565–70. 21436444 10.1126/science.1203486

[16] Oliveira G, Wu CJ. Dynamics and specificities of T cells in cancer immunotherapy. Nat Rev Cancer. 2023;23:295–316. 37046001 10.1038/s41568-023-00560-y PMC10773171

[17] Chow A, Perica K, Klebanoff CA, Wolchok JD. Clinical implications of T cell exhaustion for cancer immunotherapy. Nat Rev Clin Oncol. 2022;19:775–90. 36216928 10.1038/s41571-022-00689-z PMC10984554

[18] Wherry EJ, Kurachi M. Molecular and cellular insights into T cell exhaustion. Nat Rev Immunol. 2015;15:486–99. 26205583 10.1038/nri3862 PMC4889009

[19] Wachsmann T, Wouters AK, Remst D, Hagedoorn RS, Meeuwsen MH, van Diest E, et al. Comparing CAR and TCR engineered T cell performance as a function of tumor cell exposure. OncoImmunology. 2022;11:2033528. 35127255 10.1080/2162402X.2022.2033528 PMC8812760

[20] Galon J, Bruni D. Approaches to treat immune hot, altered and cold tumours with combination immunotherapies. Nat Rev Drug Discov. 2019;18:197–218. 30610226 10.1038/s41573-018-0007-y

[21] Hiltensperger M, Krackhardt AM. Current and future concepts for the generation and application of genetically engineered CAR-T and TCR-T cells. Front Immunol. 2023;14:1121030. 36949949 10.3389/fimmu.2023.1121030 PMC10025359

[22] Liu Y, Liu G, Wang J, Zheng Z, Jia L, Rui W, et al. Chimeric STAR receptors using TCR machinery mediate robust responses against solid tumors. Sci Transl Med. 2021;13:eabb5191. 33762437 10.1126/scitranslmed.abb5191

[23] Mansilla-Soto J, Eyquem J, Haubner S, Hamieh M, Feucht J, Paillon N, et al. HLA-independent T cell receptors for targeting tumors with low antigen density. Nat Med. 2022;28:345–52. 35027758 10.1038/s41591-021-01621-1 PMC9469647

[24] Labanieh L, Majzner RG, Mackall CL. Programming CAR-T cells to kill cancer. Nat Biomed Eng. 2018;2:377–91. 31011197 10.1038/s41551-018-0235-9

[25] Miliotou AN, Papadopoulou LC. CAR T-cell therapy: a new era in cancer immunotherapy. Curr Pharm Biotechnol. 2018;19:5–18. 29667553 10.2174/1389201019666180418095526

[26] Barros L, Couto S, da Silva Santurio D, Paixão EA, Cardoso F, da Silva VJ, et al. Systematic review of available CAR-T cell trials around the world. Cancers. 2022;14:2667. 35681646 10.3390/cancers14112667 PMC9179563

[27] Cappell KM, Kochenderfer JN. Long-term outcomes following CAR T cell therapy: what we know so far. Nat Rev Clin Oncol. 2023;20:359–71. 37055515 10.1038/s41571-023-00754-1 PMC10100620

[28] Labanieh L, Mackall CL. CAR immune cells: design principles, resistance and the next generation. Nature. 2023;614:635–48. 36813894 10.1038/s41586-023-05707-3

[29] Drougkas K, Karampinos K, Karavolias I, Koumprentziotis IA, Ploumaki I, Triantafyllou E, et al. Comprehensive clinical evaluation of CAR-T cell immunotherapy for solid tumors: a path moving forward or a dead end? J Cancer Res Clin Oncol. 2023;149:2709–34. 36564524 10.1007/s00432-022-04547-4 PMC10129996

[30] Brown CE, Badie B, Barish ME, Weng L, Ostberg JR, Chang WC, et al. Bioactivity and safety of IL13Rα2-redirected chimeric antigen receptor CD8+ T cells in patients with recurrent glioblastoma. Clin Cancer Res J Am Assoc Cancer Res. 2015;21:4062–72. 10.1158/1078-0432.CCR-15-0428 PMC4632968 26059190

[31] Brown CE, Rodriguez A, Palmer J, Ostberg JR, Naranjo A, Wagner JR, et al. Off-the-shelf, steroid-resistant, IL13Rα2-specific CAR T cells for treatment of glioblastoma. Neuro-oncology. 2022;24:1318–30. 35100373 10.1093/neuonc/noac024 PMC9340633

[32] Brown CE, Alizadeh D, Starr R, Weng L, Wagner JR, Naranjo A, et al. Regression of glioblastoma after chimeric antigen receptor T-cell therapy. N Engl J Med. 2016;375:2561–9. 28029927 10.1056/NEJMoa1610497 PMC5390684

[33] Brown CE, Hibbard JC, Alizadeh D, Blanchard MS, Natri HM, Wang D, et al. Locoregional delivery of IL-13Rα2-targeting CAR-T cells in recurrent high-grade glioma: a phase 1 trial. Nat Med. 2024;30:1001–12. 38454126 10.1038/s41591-024-02875-1 PMC11031404

[34] Vitanza NA, Johnson AJ, Wilson AL, Brown C, Yokoyama JK, Künkele A, et al. Locoregional infusion of HER2-specific CAR T cells in children and young adults with recurrent or refractory CNS tumors: an interim analysis. Nat Med. 2021;27:1544–52. 34253928 10.1038/s41591-021-01404-8

[35] Vitanza NA, Wilson AL, Huang W, Seidel K, Brown C, Gustafson JA, et al. Intraventricular B7-H3 CAR T cells for diffuse intrinsic pontine glioma: preliminary first-in-human bioactivity and safety. Cancer Discov. 2023;13:114–31. 36259971 10.1158/2159-8290.CD-22-0750 PMC9827115

[36] Ahmed N, Brawley V, Hegde M, Bielamowicz K, Kalra M, Landi D, et al. HER2-specific chimeric antigen receptor-modified virus-specific T cells for progressive glioblastoma: a phase 1 dose-escalation trial. JAMA Oncol. 2017;3:1094–101. 28426845 10.1001/jamaoncol.2017.0184 PMC5747970

[37] Goff SL, Morgan RA, Yang JC, Sherry RM, Robbins PF, Restifo NP, et al. Pilot trial of adoptive transfer of chimeric antigen receptor-transduced T cells targeting EGFRvIII in patients with glioblastoma. J Immunother. 2019;42:126–35. 30882547 10.1097/CJI.0000000000000260 PMC6691897

[38] O’Rourke DM, Nasrallah MP, Desai A, Melenhorst JJ, Mansfield K, Morrissette JJD, et al. A single dose of peripherally infused EGFRvIII-directed CAR T cells mediates antigen loss and induces adaptive resistance in patients with recurrent glioblastoma. Sci Transl Med. 2017;9:eaaa0984. 28724573 10.1126/scitranslmed.aaa0984 PMC5762203

[39] Majzner RG, Ramakrishna S, Yeom KW, Patel S, Chinnasamy H, Schultz LM, et al. GD2-CAR T cell therapy for H3K27M-mutated diffuse midline gliomas. Nature. 2022;603:934–41. 35130560 10.1038/s41586-022-04489-4 PMC8967714

[40] Choi BD, Gerstner ER, Frigault MJ, Leick MB, Mount CW, Balaj L, et al. Intraventricular CARv3-TEAM-E T cells in recurrent glioblastoma. N Engl J Med. 2024;390:1290–8. 38477966 10.1056/NEJMoa2314390 PMC11162836

[41] Chen E, Ling AL, Reardon DA, Chiocca EA. Lessons learned from phase 3 trials of immunotherapy for glioblastoma: time for longitudinal sampling? Neuro-oncology. 2024;26:211–25. 37995317 10.1093/neuonc/noad211 PMC10836778

[42] Ma S, Li X, Wang X, Cheng L, Li Z, Zhang C, et al. Current progress in CAR-T cell therapy for solid tumors. Int J Biol Sci. 2019;15:2548–60. 31754328 10.7150/ijbs.34213 PMC6854376

[43] Sterner RC, Sterner RM. CAR-T cell therapy: current limitations and potential strategies. Blood Cancer J. 2021;11:69. 33824268 10.1038/s41408-021-00459-7 PMC8024391

[44] Jogalekar MP, Rajendran RL, Khan F, Dmello C, Gangadaran P, Ahn BC. CAR T-cell-based gene therapy for cancers: new perspectives, challenges, and clinical developments. Front Immunol. 2022;13:925985. 35936003 10.3389/fimmu.2022.925985 PMC9355792

[45] Li J, Li W, Huang K, Zhang Y, Kupfer G, Zhao Q. Chimeric antigen receptor T cell (CAR-T) immunotherapy for solid tumors: lessons learned and strategies for moving forward. J Hematol Oncol. 2018;11:22. 29433552 10.1186/s13045-018-0568-6 PMC5809840

[46] Rafiq S, Hackett CS, Brentjens RJ. Engineering strategies to overcome the current roadblocks in CAR T cell therapy. Nat Rev Clin Oncol. 2020;17:147–67. 31848460 10.1038/s41571-019-0297-y PMC7223338

[47] Daei Sorkhabi A, Mohamed Khosroshahi L, Sarkesh A, Mardi A, Aghebati-Maleki A, Aghebati-Maleki L, et al. The current landscape of CAR T-cell therapy for solid tumors: mechanisms, research progress, challenges, and counterstrategies. Front Immunol. 2023;14:1113882. 37020537 10.3389/fimmu.2023.1113882 PMC10067596

[48] Hong M, Talluri S, Chen YY. Advances in promoting chimeric antigen receptor T cell trafficking and infiltration of solid tumors. Curr Opin Biotechnol. 2023;84:103020. 37976958 10.1016/j.copbio.2023.103020

[49] Hawkins ER, D’Souza RR, Klampatsa A. Armored CAR T-cells: the next chapter in T-cell cancer immunotherapy. Biol Targets Ther. 2021;15:95–105. 10.2147/BTT.S291768 PMC8053711 33883875

[50] Han D, Xu Z, Zhuang Y, Ye Z, Qian Q. Current progress in CAR-T cell therapy for hematological malignancies. J Cancer. 2021;12:326–34. 33391429 10.7150/jca.48976 PMC7738987

[51] Sang W, Shi M, Yang J, Cao J, Xu L, Yan D, et al. Phase II trial of co‐administration of CD19‐ and CD20‐targeted chimeric antigen receptor T cells for relapsed and refractory diffuse large B cell lymphoma. Cancer Med. 2020;9:5827–38. 32608579 10.1002/cam4.3259 PMC7433814

[52] Muhammad N, Wang R, Li W, Zhang Z, Chang Y, Hu Y, et al. A novel TanCAR targeting IL13Rα2 and EphA2 for enhanced glioblastoma therapy. Mol Ther Oncolytics. 2022;24:729–41. 35317513 10.1016/j.omto.2022.02.012 PMC8908045

[53] Wing A, Fajardo CA, Posey AD Jr, Shaw C, Da T, Young RM, et al. Improving CART-cell therapy of solid tumors with oncolytic virus–driven production of a bispecific T-cell engager. Cancer Immunol Res. 2018;6:605–16. 29588319 10.1158/2326-6066.CIR-17-0314 PMC6688490

[54] Weller M, Butowski N, Tran DD, Recht LD, Lim M, Hirte H, et al. Rindopepimut with temozolomide for patients with newly diagnosed, EGFRvIII-expressing glioblastoma (ACT IV): a randomised, double-blind, international phase 3 trial. Lancet Oncol. 2017;18:1373–85. 28844499 10.1016/S1470-2045(17)30517-X

[55] Lohmueller JJ, Ham JD, Kvorjak M, Finn OJ. mSA2 affinity-enhanced biotin-binding CAR T cells for universal tumor targeting. Oncoimmunology. 2018;7:e1368604. 10.1080/2162402X.2017.1368604 PMC5739565 29296519

[56] Cho JH, Collins JJ, Wong WW. Universal chimeric antigen receptors for multiplexed and logical control of T cell responses. Cell. 2018;173:1426–38. 29706540 10.1016/j.cell.2018.03.038 PMC5984158

[57] Reinhard K, Rengstl B, Oehm P, Michel K, Billmeier A, Hayduk N, et al. An RNA vaccine drives expansion and efficacy of claudin-CAR-T cells against solid tumors. Science. 2020;367:446–53. 31896660 10.1126/science.aay5967

[58] Ma L, Hostetler A, Morgan DM, Maiorino L, Sulkaj I, Whittaker CA, et al. Vaccine-boosted CAR T crosstalk with host immunity to reject tumors with antigen heterogeneity. Cell. 2023;186:3148–65. 37413990 10.1016/j.cell.2023.06.002 PMC10372881

[59] Mackensen A, Haanen J, Koenecke C, Alsdorf W, Wagner-Drouet E, Borchmann P, et al. CLDN6-specific CAR-T cells plus amplifying RNA vaccine in relapsed or refractory solid tumors: the phase 1 BNT211-01 trial. Nat Med. 2023;29:2844–53. 37872225 10.1038/s41591-023-02612-0 PMC10667102

[60] Märkl F, Huynh D, Endres S, Kobold S. Utilizing chemokines in cancer immunotherapy. Trends Cancer. 2022;8:670–82. 35501268 10.1016/j.trecan.2022.04.001

[61] Neurath MF. Targeting immune cell circuits and trafficking in inflammatory bowel disease. Nat Immunol. 2019;20:970–9. 31235952 10.1038/s41590-019-0415-0

[62] Jin L, Tao H, Karachi A, Long Y, Hou AY, Na M, et al. CXCR1- or CXCR2-modified CAR T cells co-opt IL-8 for maximal antitumor efficacy in solid tumors. Nat Commun. 2019;10:4016. 31488817 10.1038/s41467-019-11869-4 PMC6728370

[63] Yoshie O, Matsushima K. CCR4 and its ligands: from bench to bedside. Int Immunol. 2015;27:11–20. 25087232 10.1093/intimm/dxu079

[64] Di Stasi A, De Angelis B, Rooney CM, Zhang L, Mahendravada A, Foster AE, et al. T lymphocytes coexpressing CCR4 and a chimeric antigen receptor targeting CD30 have improved homing and antitumor activity in a Hodgkin tumor model. Blood. 2009;113:6392–402. 19377047 10.1182/blood-2009-03-209650 PMC2710932

[65] Grover NS, Ivanova A, Moore DT, Cheng CJA, Babinec C, West J, et al. CD30-directed CAR-T cells co-expressing CCR4 in relapsed/refractory hodgkin lymphoma and CD30+ cutaneous T cell lymphoma. Blood. 2021;138:742–742.

[66] Craddock JA, Lu A, Bear A, Pule M, Brenner MK, Rooney CM, et al. Enhanced tumor trafficking of GD2 chimeric antigen receptor T cells by expression of the chemokine receptor CCR2b. J Immunother. 2010;33:780–8. 20842059 10.1097/CJI.0b013e3181ee6675 PMC2998197

[67] Moon EK, Carpenito C, Sun J, Wang LC, Kapoor V, Predina J, et al. Expression of a functional CCR2 receptor enhances tumor localization and tumor eradication by retargeted human T cells expressing a mesothelin-specific chimeric antibody receptor. Clin Cancer Res. 2011;17:4719–30. 21610146 10.1158/1078-0432.CCR-11-0351 PMC3612507

[68] Li G, Zhang Q, Han Z, Zhu Y, Shen H, Liu Z, et al. IL-7 and CCR2b co-expression-mediated enhanced CAR-T survival and infiltration in solid tumors. Front Oncol. 2021;11:734593. 34778046 10.3389/fonc.2021.734593 PMC8579717

[69] Wang Y, Wang J, Yang X, Yang J, Lu P, Zhao L, et al. Chemokine receptor CCR2b enhanced anti-tumor function of chimeric antigen receptor T cells targeting mesothelin in a non-small-cell lung carcinoma model. Front Immunol. 2021;12:628906. 33777013 10.3389/fimmu.2021.628906 PMC7992009

[70] Luo H, Su J, Sun R, Sun Y, Wang Y, Dong Y, et al. Coexpression of IL7 and CCL21 increases efficacy of CAR-T cells in solid tumors without requiring preconditioned lymphodepletion. Clin Cancer Res. 2020;26:5494–505. 32816947 10.1158/1078-0432.CCR-20-0777

[71] Sasaki T, Sakoda Y, Adachi K, Tokunaga Y, Tamada K. Therapeutic effects of anti‐GM2 CAR‐T cells expressing IL‐7 and CCL19 for GM2‐positive solid cancer in xenograft model. Cancer Med. 2023;12:12569–80. 37031457 10.1002/cam4.5907 PMC10278466

[72] Trinh T, Adams WA, Calescibetta A, Tu N, Dalton R, So T, et al. CX3CR1 deficiency-induced TIL tumor restriction as a novel addition for CAR-T design in solid malignancies. iScience. 2023;26:106443. 37070068 10.1016/j.isci.2023.106443 PMC10105289

[73] Tchou J, Zhao Y, Levine BL, Zhang PJ, Davis MM, Melenhorst JJ, et al. Safety and efficacy of intratumoral injections of chimeric antigen receptor (CAR) T cells in metastatic breast cancer. Cancer Immunol Res. 2017;5:1152–61. 29109077 10.1158/2326-6066.CIR-17-0189 PMC5712264

[74] Hardaway JC, Prince E, Arepally A, Katz SC. Regional infusion of chimeric antigen receptor T cells to overcome barriers for solid tumor immunotherapy. J Vasc Interv Radiol. 2018;29:1017–21. 29935783 10.1016/j.jvir.2018.03.001

[75] Prapa M, Chiavelli C, Golinelli G, Grisendi G, Bestagno M, Di Tinco R, et al. GD2 CAR T cells against human glioblastoma. NPJ Precis Oncol. 2021;5:93. 34707200 10.1038/s41698-021-00233-9 PMC8551169

[76] Katz SC, Burga RA, McCormack E, Wang LJ, Mooring W, Point GR, et al. Phase I hepatic immunotherapy for metastases study of intra-arterial chimeric antigen receptor–modified T-cell therapy for CEA+ liver metastases. Clin Cancer Res. 2015;21:3149–59. 25850950 10.1158/1078-0432.CCR-14-1421 PMC4506253

[77] Katz SC, Hardaway J, Prince E, Guha P, Cunetta M, Moody A, et al. HITM-SIR: phase Ib trial of intraarterial chimeric antigen receptor T-cell therapy and selective internal radiation therapy for CEA+ liver metastases. Cancer Gene Ther. 2020;27:341–55. 31155611 10.1038/s41417-019-0104-z

[78] Tran E, Chinnasamy D, Yu Z, Morgan RA, Lee CC, Restifo NP, et al. Immune targeting of fibroblast activation protein triggers recognition of multipotent bone marrow stromal cells and cachexia, Immune targeting of fibroblast activation protein triggers recognition of multipotent bone marrow stromal cells and cachexia. J Exp Med. 2013;210:1125–35. 23712432 10.1084/jem.20130110 PMC3674706

[79] Wang LC, Lo A, Scholler J, Sun J, Majumdar RS, Kapoor V, et al. Targeting fibroblast activation protein in tumor stroma with chimeric antigen receptor T cells can inhibit tumor growth and augment host immunity without severe toxicity. Cancer Immunol Res. 2014;2:154–66. 24778279 10.1158/2326-6066.CIR-13-0027 PMC4007316

[80] Caruana I, Savoldo B, Hoyos V, Weber G, Liu H, Kim ES, et al. Heparanase promotes tumor infiltration and antitumor activity of CAR-redirected T lymphocytes. Nat Med. 2015;21:524–9. 25849134 10.1038/nm.3833 PMC4425589

[81] Wang S, Li Y, Xu C, Dong J, Wei J. An oncolytic vaccinia virus encoding hyaluronidase reshapes the extracellular matrix to enhance cancer chemotherapy and immunotherapy. J Immunother Cancer. 2024;12:e008431. 38458640 10.1136/jitc-2023-008431 PMC10921532

[82] Hingorani SR, Zheng L, Bullock AJ, Seery TE, Harris WP, Sigal DS, et al. HALO 202: randomized phase II study of PEGPH20 plus nab-paclitaxel/gemcitabine versus nab-paclitaxel/gemcitabine in patients with untreated, metastatic pancreatic ductal adenocarcinoma. J Clin Oncol. 2018;36:359–66. 29232172 10.1200/JCO.2017.74.9564

[83] Ramanathan RK, McDonough SL, Philip PA, Hingorani SR, Lacy J, Kortmansky JS, et al. Phase IB/II randomized study of FOLFIRINOX plus pegylated recombinant human hyaluronidase versus FOLFIRINOX alone in patients with metastatic pancreatic adenocarcinoma: SWOG S1313. J Clin Oncol. 2019;37:1062–9. 30817250 10.1200/JCO.18.01295 PMC6494359

[84] Mehrabadi AZ, Ranjbar R, Farzanehpour M, Shahriary A, Dorostkar R, Hamidinejad MA, et al. Therapeutic potential of CAR T cell in malignancies: a scoping review. Biomed Pharmacother. 2022;146:112512. 34894519 10.1016/j.biopha.2021.112512

[85] Marofi F, Motavalli R, Safonov VA, Thangavelu L, Yumashev AV, Alexander M, et al. CAR T cells in solid tumors: challenges and opportunities. Stem Cell Res Ther. 2021;12:81. 33494834 10.1186/s13287-020-02128-1 PMC7831265

[86] Zhang H, Dai Z, Wu W, Wang Z, Zhang N, Zhang L, et al. Regulatory mechanisms of immune checkpoints PD-L1 and CTLA-4 in cancer. J Exp Clin Cancer Res. 2021;40:184. 34088360 10.1186/s13046-021-01987-7 PMC8178863

[87] Kornepati AVR, Vadlamudi RK, Curiel TJ. Programmed death ligand 1 signals in cancer cells. Nat Rev Cancer. 2022;22:174–89. 35031777 10.1038/s41568-021-00431-4 PMC9989967

[88] Abdoli Shadbad M, Hemmat N, Khaze Shahgoli V, Derakhshani A, Baradaran F, Brunetti O, et al. A systematic review on PD-1 blockade and PD-1 gene-editing of CAR-T cells for glioma therapy: from deciphering to personalized medicine. Front Immunol. 2022;12:788211. 35126356 10.3389/fimmu.2021.788211 PMC8807490

[89] Gray KD, McCloskey JE, Vedvyas Y, Kalloo OR, Eshaky SE, Yang Y, et al. PD1 blockade enhances ICAM1-directed CAR T therapeutic efficacy in advanced thyroid cancer. Clin Cancer Res. 2020;26:6003–16. 32887724 10.1158/1078-0432.CCR-20-1523 PMC7709864

[90] Li P, Yang L, Li T, Bin S, Sun B, Huang Y, et al. The third generation anti-HER2 chimeric antigen receptor mouse T cells alone or together with anti-PD1 antibody inhibits the growth of mouse breast tumor cells expressing HER2 in vitro and in immune competent mice. Front Oncol. 2020;10:1143. 32766150 10.3389/fonc.2020.01143 PMC7381237

[91] Jaspers JE, Khan JF, Godfrey WD, Lopez AV, Ciampricotti M, Rudin CM, et al. IL-18–secreting CAR T cells targeting DLL3 are highly effective in small cell lung cancer models. J Clin Investig. 2023;133:e166028. 36951942 10.1172/JCI166028 PMC10145930

[92] Adusumilli PS, Zauderer MG, Rivière I, Solomon SB, Rusch VW, O'Cearbhaill RE, et al. A phase I trial of regional mesothelin-targeted CAR T-cell therapy in patients with malignant pleural disease, in combination with the anti–PD-1 agent pembrolizumab. Cancer Discov. 2021;11:2748–63. 34266984 10.1158/2159-8290.CD-21-0407 PMC8563385

[93] Wang Z, Li N, Feng K, Chen M, Zhang Y, Liu Y, et al. Phase I study of CAR-T cells with PD-1 and TCR disruption in mesothelin-positive solid tumors. Cell Mol Immunol. 2021;18:2188–98. 34381179 10.1038/s41423-021-00749-x PMC8429583

[94] Agarwal S, Aznar MA, Rech AJ, Good CR, Kuramitsu S, Da T, et al. Deletion of the inhibitory co-receptor CTLA-4 enhances and invigorates chimeric antigen receptor T cells. Immunity. 2023;56:2388–407. 37776850 10.1016/j.immuni.2023.09.001 PMC10591801

[95] Cheng K, Feng X, Chai Z, Wang Z, Liu Z, Yan Z, et al. 4-1BB-based CAR T cells effectively reverse exhaustion and enhance the anti-tumor immune response through autocrine PD-L1 scFv antibody. Int J Mol Sci. 2023;24:4197. 36835603 10.3390/ijms24044197 PMC9961031

[96] Dunn ZS, Qu Y, MacMullan M, Chen X, Cinay G, Wang P. Secretion of 4-1BB ligand crosslinked to PD-1 checkpoint inhibitor potentiates chimeric antigen receptor T cell solid tumor efficacy. Hum Gene Ther. 2023. 10.1089/hum.2022.068. 10.1089/hum.2022.068 36851890

[97] Sun C, Wang B, Hao S. Adenosine-A2A receptor pathway in cancer immunotherapy. Front Immunol. 2022;13:837230. 35386701 10.3389/fimmu.2022.837230 PMC8977492

[98] Masoumi E, Jafarzadeh L, Mirzaei HR, Alishah K, Fallah-Mehrjardi K, Rostamian H, et al. Genetic and pharmacological targeting of A2a receptor improves function of anti-mesothelin CAR T cells. J Exp Clin Cancer Res. 2020;39:49. 32151275 10.1186/s13046-020-01546-6 PMC7063771

[99] Klysz DD, Fowler C, Malipatlolla M, Stuani L, Freitas KA, Chen Y, et al. Inosine induces stemness features in CAR-T cells and enhances potency. Cancer Cell. 2024:S1535610824000084. 10.1016/j.ccell.2024.01.002. 10.1016/j.ccell.2024.01.002 PMC10923096 38278150

[100] Wang Z, Liu Q, Risu N, Fu J, Zou Y, Tang J, et al. Galunisertib enhances chimeric antigen receptor-modified T cell function. Eur J Histochem. 2020;64:3122. 32705856 10.4081/ejh.2020.3122 PMC7388644

[101] Alabanza LM, Xiong Y, Vu B, Webster B, Wu D, Hu P, et al. Armored BCMA CAR T cells eliminate multiple myeloma and are resistant to the suppressive effects of TGF-β. Front Immunol. 2022;13:832645. 35222421 10.3389/fimmu.2022.832645 PMC8863610

[102] Qin L, Cui Y, Yuan T, Chen D, Zhao R, Li S, et al. Co-expression of a PD-L1-specific chimeric switch receptor augments the efficacy and persistence of CAR T cells via the CD70-CD27 axis. Nat Commun. 2022;13:6051. 36229619 10.1038/s41467-022-33793-w PMC9561169

[103] Sukumaran S, Watanabe N, Bajgain P, Raja K, Mohammed S, Fisher WE, et al. Enhancing the potency and specificity of engineered T cells for cancer treatment. Cancer Discov. 2018;8:972–87. 29880586 10.1158/2159-8290.CD-17-1298 PMC6428579

[104] June CH, O’Connor RS, Kawalekar OU, Ghassemi S, Milone MC. CAR T cell immunotherapy for human cancer. Science. 2018;359:1361–5. 29567707 10.1126/science.aar6711

[105] Bell M, Gottschalk S. Engineered cytokine signaling to improve CAR T cell effector function. Front Immunol. 2021;12:684642. 34177932 10.3389/fimmu.2021.684642 PMC8220823

[106] Sauer T, Parikh K, Sharma S, Omer B, Sedloev D, Chen Q, et al. CD70-specific CAR T cells have potent activity against acute myeloid leukemia without HSC toxicity. Blood. 2021;138:318–30. 34323938 10.1182/blood.2020008221 PMC8323977

[107] Panowski SH, Srinivasan S, Tan N, Tacheva-Grigorova SK, Smith B, Mak Y, et al. Preclinical development and evaluation of allogeneic CAR T cells targeting CD70 for the treatment of renal cell carcinoma. Cancer Res. 2022;82:2610–24. 35294525 10.1158/0008-5472.CAN-21-2931

[108] Smirnov S, Mateikovich P, Samochernykh K, Shlyakhto E. Recent advances on CAR-T signaling pave the way for prolonged persistence and new modalities in clinic. Front Immunol. 2024;15:1335424. 38455066 10.3389/fimmu.2024.1335424 PMC10918004

[109] Wyatt MM, Huff LW, Nelson MH, Neal LR, Medvec AR, Rangel Rivera GO, et al. Augmenting TCR signal strength and ICOS costimulation results in metabolically fit and therapeutically potent human CAR Th17 cells. Mol Ther. 2023;31:2120–31. 37081789 10.1016/j.ymthe.2023.04.010 PMC10362414

[110] Guedan S, Posey AD Jr, Shaw C, Wing A, Da T, Patel PR, et al. Enhancing CAR T cell persistence through ICOS and 4-1BB costimulation. JCI Insight. 2018;3:e96976. 29321369 10.1172/jci.insight.96976 PMC5821198

[111] Moreno-Cortes E, Franco-Fuquen P, Garcia-Robledo JE, Forero J, Booth N, Castro JE. ICOS and OX40 tandem co-stimulation enhances CAR T-cell cytotoxicity and promotes T-cell persistence phenotype. Front Oncol. 2023;13:1200914. 37719008 10.3389/fonc.2023.1200914 PMC10502212

[112] Chabannon C, Bonini C. Structure of and signalling through chimeric antigen receptor. In: Kröger N, Gribben J, Chabannon C, Yakoub-Agha I, Einsele H, editors. The EBMT/EHA CAR-T cell handbook. Cham: Springer International Publishing; 2022. p. 3–5. 36122078

[113] Li K, Xu J, Wang J, Lu C, Dai Y, Dai Q, et al. Dominant-negative transforming growth factor-β receptor-armoured mesothelin-targeted chimeric antigen receptor T cells slow tumour growth in a mouse model of ovarian cancer. Cancer Immunol Immunother. 2023;72:917–28. 36166071 10.1007/s00262-022-03290-6 PMC10025183

[114] Rana PS, Murphy EV, Kort J, Driscoll JJ. Road testing new CAR design strategies in multiple myeloma. Front Immunol. 2022;13:957157. 36016950 10.3389/fimmu.2022.957157 PMC9395635

[115] Tang L, Pan S, Wei X, Xu X, Wei Q. Arming CAR-T cells with cytokines and more: innovations in the fourth-generation CAR-T development. Mol Ther. 2023;31:3146–62. 37803832 10.1016/j.ymthe.2023.09.021 PMC10638038

[116] Agliardi G, Liuzzi AR, Hotblack A, De Feo D, Núñez N, Stowe CL, et al. Intratumoral IL-12 delivery empowers CAR-T cell immunotherapy in a pre-clinical model of glioblastoma. Nat Commun. 2021;12:444. 33469002 10.1038/s41467-020-20599-x PMC7815781

[117] Lee E, Murad JP, Christian L, Gibson J, Yamaguchi Y, Cullen C, et al. Antigen-dependent IL-12 signaling in CAR T cells promotes regional to systemic disease targeting. Nat Commun. 2023;14:4737. 37550294 10.1038/s41467-023-40115-1 PMC10406808

[118] Hu B, Ren J, Luo Y, Keith B, Young RM, Scholler J, et al. Augmentation of antitumor immunity by human and mouse CAR T cells secreting IL-18. Cell Rep. 2017;20:3025–33. 28954221 10.1016/j.celrep.2017.09.002 PMC6002762

[119] Waldmann TA, Dubois S, Miljkovic MD, Conlon KC. IL-15 in the combination immunotherapy of cancer. Front Immunol. 2020;11:868. 32508818 10.3389/fimmu.2020.00868 PMC7248178

[120] Zannikou M, Duffy JT, Levine RN, Seblani M, Liu Q, Presser A, et al. IL15 modification enables CAR T cells to act as a dual targeting agent against tumor cells and myeloid-derived suppressor cells in GBM. J Immunother Cancer. 2023;11:e006239. 36759014 10.1136/jitc-2022-006239 PMC9923337

[121] Zhou Y, Farooq MA, Ajmal I, He C, Gao Y, Guo D, et al. Co-expression of IL-4/IL-15-based inverted cytokine receptor in CAR-T cells overcomes IL-4 signaling in immunosuppressive pancreatic tumor microenvironment. Biomed Pharmacother. 2023;168:115740. 37865999 10.1016/j.biopha.2023.115740

[122] Zhao Y, Chen J, Andreatta M, Feng B, Xie YQ, Wenes M, et al. IL-10-expressing CAR T cells resist dysfunction and mediate durable clearance of solid tumors and metastases. Nat Biotechnol. 2024. 10.1038/s41587-023-02060-8. 10.1038/s41587-023-02060-8 38168996

[123] Wang D, Prager BC, Gimple RC, Aguilar B, Alizadeh D, Tang H, et al. CRISPR screening of CAR T cells and cancer stem cells reveals critical dependencies for cell-based therapies. Cancer Discov. 2021;11:1192–211. 33328215 10.1158/2159-8290.CD-20-1243 PMC8406797

[124] Zhou P, Shi H, Huang H, Sun X, Yuan S, Chapman NM, et al. Single-cell CRISPR screens in vivo map T cell fate regulomes in cancer. Nature. 2023;624:154–63. 37968405 10.1038/s41586-023-06733-x PMC10700132

[125] Zheng W, Wei J, Zebley CC, Jones LL, Dhungana Y, Wang YD, et al. Regnase-1 suppresses TCF-1+ precursor exhausted T-cell formation to limit CAR–T-cell responses against ALL. Blood. 2021;138:122–35. 33690816 10.1182/blood.2020009309 PMC8288655

[126] Dong MB, Wang G, Chow RD, Ye L, Zhu L, Dai X, et al. Systematic immunotherapy target discovery using genome-scale in vivo CRISPR screens in CD8 T cells. Cell. 2019;178:1189–204. 31442407 10.1016/j.cell.2019.07.044 PMC6719679

[127] Carnevale J, Shifrut E, Kale N, Nyberg WA, Blaeschke F, Chen YY, et al. RASA2 ablation in T cells boosts antigen sensitivity and long-term function. Nature. 2022;609:174–82. 36002574 10.1038/s41586-022-05126-w PMC9433322

[128] Ye L, Park JJ, Peng L, Yang Q, Chow RD, Dong MB, et al. A genome-scale gain-of-function CRISPR screen in CD8 T cells identifies proline metabolism as a means to enhance CAR-T therapy. Cell Metab. 2022;34:595–614. 35276062 10.1016/j.cmet.2022.02.009 PMC8986623

[129] Legut M, Gajic Z, Guarino M, Daniloski Z, Rahman JA, Xue X, et al. A genome-scale screen for synthetic drivers of T cell proliferation. Nature. 2022;603:728–35. 35296855 10.1038/s41586-022-04494-7 PMC9908437

[130] Morris EC, Neelapu SS, Giavridis T, Sadelain M. Cytokine release syndrome and associated neurotoxicity in cancer immunotherapy. Nat Rev Immunol. 2022;22:85–96. 34002066 10.1038/s41577-021-00547-6 PMC8127450

[131] Lee DW, Gardner R, Porter DL, Louis CU, Ahmed N, Jensen M, et al. Current concepts in the diagnosis and management of cytokine release syndrome. Blood. 2014;124:188–95. 24876563 10.1182/blood-2014-05-552729 PMC4093680

[132] Norelli M, Camisa B, Barbiera G, Falcone L, Purevdorj A, Genua M, et al. Monocyte-derived IL-1 and IL-6 are differentially required for cytokine-release syndrome and neurotoxicity due to CAR T cells. Nat Med. 2018;24:739–48. 29808007 10.1038/s41591-018-0036-4

[133] Shimabukuro-Vornhagen A, Gödel P, Subklewe M, Stemmler HJ, Schlößer HA, Schlaak M, et al. Cytokine release syndrome. J Immunother Cancer. 2018;6:56. 29907163 10.1186/s40425-018-0343-9 PMC6003181

[134] Pennisi M, Jain T, Santomasso BD, Mead E, Wudhikarn K, Silverberg ML, et al. Comparing CAR T-cell toxicity grading systems: application of the ASTCT grading system and implications for management. Blood Adv. 2020;4:676–86. 32084260 10.1182/bloodadvances.2019000952 PMC7042979

[135] Kowolik CM, Topp MS, Gonzalez S, Pfeiffer T, Olivares S, Gonzalez N, et al. CD28 costimulation provided through a CD19-specific chimeric antigen receptor enhances in vivo persistence and antitumor efficacy of adoptively transferred T cells. Cancer Res. 2006;66:10995–1004. 17108138 10.1158/0008-5472.CAN-06-0160

[136] Brentjens RJ, Latouche JB, Santos E, Marti F, Gong MC, Lyddane C, et al. Eradication of systemic B-cell tumors by genetically targeted human T lymphocytes co-stimulated by CD80 and interleukin-15. Nat Med. 2003;9:279–86. 12579196 10.1038/nm827

[137] Imai C, Mihara K, Andreansky M, Nicholson IC, Pui CH, Geiger TL, et al. Chimeric receptors with 4-1BB signaling capacity provoke potent cytotoxicity against acute lymphoblastic leukemia. Leukemia. 2004;18:676–84. 14961035 10.1038/sj.leu.2403302

[138] Giavridis T, van der Stegen S, Eyquem J, Hamieh M, Piersigilli A, Sadelain M. CAR T cell-induced cytokine release syndrome is mediated by macrophages and abated by IL-1 blockade. Nat Med. 2018;24:731–8. 29808005 10.1038/s41591-018-0041-7 PMC6410714

[139] Faulhaber LD, Phuong AQ, Hartsuyker KJ, Cho Y, Mand KK, Harper SD, et al. Brain capillary obstruction during neurotoxicity in a mouse model of anti-CD19 chimeric antigen receptor T-cell therapy. Brain Commun. 2022;4:fcab309. 35169706 10.1093/braincomms/fcab309 PMC8833245

[140] Vinnakota JM, Biavasco F, Schwabenland M, Chhatbar C, Adams RC, Erny D, et al. Targeting TGFβ-activated kinase-1 activation in microglia reduces CAR T immune effector cell-associated neurotoxicity syndrome. Nat Cancer. 2024. 10.1038/s43018-024-00764-7. 10.1038/s43018-024-00764-7 38741011

[141] Lee DW, Santomasso BD, Locke FL, Ghobadi A, Turtle CJ, Brudno JN, et al. ASTCT consensus grading for cytokine release syndrome and neurologic toxicity associated with immune effector cells. Biol Blood Marrow Transplant J Am Soc Blood Marrow Transplant. 2019;25:625–38. 10.1016/j.bbmt.2018.12.758 PMC12180426 30592986

[142] Strati P, Ahmed S, Furqan F, Fayad LE, Lee HJ, Iyer SP, et al. Prognostic impact of corticosteroids on efficacy of chimeric antigen receptor T-cell therapy in large B-cell lymphoma. Blood. 2021;137:3272–6. 33534891 10.1182/blood.2020008865 PMC8351896

[143] Choe JH, Watchmaker PB, Simic MS, Gilbert RD, Li AW, Krasnow NA, et al. SynNotch-CAR T cells overcome challenges of specificity, heterogeneity, and persistence in treating glioblastoma. Sci Transl Med. 2021;13:eabe7378. 33910979 10.1126/scitranslmed.abe7378 PMC8362330

[144] Kloss CC, Condomines M, Cartellieri M, Bachmann M, Sadelain M. Combinatorial antigen recognition with balanced signaling promotes selective tumor eradication by engineered T cells. Nat Biotechnol. 2013;31:71–75. 23242161 10.1038/nbt.2459 PMC5505184

[145] Fedorov VD, Themeli M, Sadelain M. PD-1- and CTLA-4-based inhibitory chimeric antigen receptors (iCARs) divert off-target immunotherapy responses. Sci Transl Med. 2013;5:215ra172. 24337479 10.1126/scitranslmed.3006597 PMC4238416

[146] Richman SA, Wang LC, Moon EK, Khire UR, Albelda SM, Milone MC, et al. Ligand-induced degradation of a CAR permits reversible remote control of CAR T cell activity in vitro and in vivo. Mol Ther J Am Soc Gene Ther. 2020;28:1600–13. 10.1016/j.ymthe.2020.06.004 PMC7335755 32559430

[147] Giordano-Attianese G, Gainza P, Gray-Gaillard E, Cribioli E, Shui S, Kim S, et al. A computationally designed chimeric antigen receptor provides a small-molecule safety switch for T-cell therapy. Nat Biotechnol. 2020;38:426–32. 32015549 10.1038/s41587-019-0403-9

[148] Juillerat A, Tkach D, Busser BW, Temburni S, Valton J, Duclert A, et al. Modulation of chimeric antigen receptor surface expression by a small molecule switch. BMC Biotechnol. 2019;19:44. 31269942 10.1186/s12896-019-0537-3 PMC6610870

[149] Zajc CU, Dobersberger M, Schaffner I, Mlynek G, Pühringer D, Salzer B, et al. A conformation-specific ON-switch for controlling CAR T cells with an orally available drug. Proc Natl Acad Sci USA. 2020;117:14926–35. 32554495 10.1073/pnas.1911154117 PMC7334647

[150] Wu C-Y, Roybal KT, Puchner EM, Onuffer J, Lim WA. Remote control of therapeutic T cells through a small molecule-gated chimeric receptor. Science. 2015;350:aab4077. 26405231 10.1126/science.aab4077 PMC4721629

[151] Labanieh L, Majzner RG, Klysz D, Sotillo E, Fisher CJ, Vilches-Moure JG, et al. Enhanced safety and efficacy of protease-regulated CAR-T cell receptors. Cell. 2022;185:1745–63. 35483375 10.1016/j.cell.2022.03.041 PMC9467936

[152] Wang X, Meng F, Li X, Xue L, Chen A, Qiu Y, et al. Nanomodified switch induced precise and moderate activation of CAR-T cells for solid tumors. Adv Sci. 2023;10:e2205044. 10.1002/advs.202205044 PMC10131841 36755195

[153] Wolf NK, Kissiov DU, Raulet DH. Roles of natural killer cells in immunity to cancer, and applications to immunotherapy. Nat Rev Immunol. 2023;23:90–105. 35637393 10.1038/s41577-022-00732-1

[154] Chan CJ, Smyth MJ, Martinet L. Molecular mechanisms of natural killer cell activation in response to cellular stress. Cell Death Differ. 2014;21:5–14. 23579243 10.1038/cdd.2013.26 PMC3857624

[155] Vivier E, Tomasello E, Baratin M, Walzer T, Ugolini S. Functions of natural killer cells. Nat Immunol. 2008;9:503–10. 18425107 10.1038/ni1582

[156] Shifrin N, Raulet DH, Ardolino M. NK cell self tolerance, responsiveness and missing self recognition. Semin Immunol. 2014;26:138–44. 24629893 10.1016/j.smim.2014.02.007 PMC3984600

[157] Joncker NT, Fernandez NC, Treiner E, Vivier E, Raulet DH. NK cell responsiveness is tuned commensurate with the number of inhibitory receptors for self-MHC class I: the rheostat model. J Immunol. 2009;182:4572–80. 19342631 10.4049/jimmunol.0803900 PMC2938179

[158] Ljunggren H-G, Kärre K. In search of the ‘missing self’: MHC molecules and NK cell recognition. Immunol Today. 1990;11:237–44. 2201309 10.1016/0167-5699(90)90097-s

[159] Anegón I, Cuturi MC, Trinchieri G, Perussia B. Interaction of Fc receptor (CD16) ligands induces transcription of interleukin 2 receptor (CD25) and lymphokine genes and expression of their products in human natural killer cells. J Exp Med. 1988;167:452–72. 2831292 10.1084/jem.167.2.452 PMC2188858

[160] Ochoa MC, Minute L, Rodriguez I, Garasa S, Perez-Ruiz E, Inogés S, et al. Antibody‐dependent cell cytotoxicity: immunotherapy strategies enhancing effector NK cells. Immunol Cell Biol. 2017;95:347–55. 28138156 10.1038/icb.2017.6

[161] Orange JS. Formation and function of the lytic NK-cell immunological synapse. Nat Rev Immunol. 2008;8:713–25. 19172692 10.1038/nri2381 PMC2772177

[162] Topham NJ, Hewitt EW. Natural killer cell cytotoxicity: how do they pull the trigger? Immunology. 2009;128:7–15. 19689731 10.1111/j.1365-2567.2009.03123.x PMC2747134

[163] Prager I, Liesche C, van Ooijen H, Urlaub D, Verron Q, Sandström N, et al. NK cells switch from granzyme B to death receptor-mediated cytotoxicity during serial killing. J Exp Med. 2019;216:2113–27. 31270246 10.1084/jem.20181454 PMC6719417

[164] Crouse J, Xu HC, Lang PA, Oxenius A. NK cells regulating T cell responses: mechanisms and outcome. Trends Immunol. 2015;36:49–58. 25432489 10.1016/j.it.2014.11.001

[165] Hercend T, Farace F, Baume D, Charpentier F, Droz JP, Triebel F, et al. Immunotherapy with lymphokine-activated natural killer cells and recombinant interleukin-2: a feasibility trial in metastatic renal cell carcinoma. J Biol Response Mod. 1990;9:546–55. 2074441

[166] Berrien-Elliott MM, Jacobs MT, Fehniger TA. Allogeneic natural killer cell therapy. Blood. 2023;141:856–68. 36416736 10.1182/blood.2022016200 PMC10023727

[167] Mitra A, Barua A, Huang L, Ganguly S, Feng Q, He B. From bench to bedside: the history and progress of CAR T cell therapy. Front Immunol. 2023;14:1188049. 37256141 10.3389/fimmu.2023.1188049 PMC10225594

[168] Bald T, Krummel MF, Smyth MJ, Barry KC. The NK cell-cancer cycle: advances and new challenges in NK cell-based immunotherapies. Nat Immunol. 2020;21:835–47. 32690952 10.1038/s41590-020-0728-z PMC8406687

[169] Cózar B, Greppi M, Carpentier S, Narni-Mancinelli E, Chiossone L, Vivier E. Tumor-infiltrating natural killer cells. Cancer Discov. 2021;11:34–44. 33277307 10.1158/2159-8290.CD-20-0655 PMC7611243

[170] Peng L-S, Zhang JY, Teng YS, Zhao YL, Wang TT, Mao FY, et al. Tumor-associated monocytes/macrophages impair NK-cell function via TGFβ1 in human gastric cancer. Cancer Immunol Res. 2017;5:248–56. 28148545 10.1158/2326-6066.CIR-16-0152

[171] Zhang Q-F, Yin WW, Xia Y, Yi YY, He QF, Wang X, et al. Liver-infiltrating CD11b-CD27- NK subsets account for NK-cell dysfunction in patients with hepatocellular carcinoma and are associated with tumor progression. Cell Mol Immunol. 2017;14:819–29. 27321064 10.1038/cmi.2016.28 PMC5649104

[172] Zheng X, Qian Y, Fu B, Jiao D, Jiang Y, Chen P, et al. Mitochondrial fragmentation limits NK cell-based tumor immunosurveillance. Nat Immunol. 2019;20:1656–67. 31636463 10.1038/s41590-019-0511-1

[173] Garcia-Chagollan M, Carranza-Torres IE, Carranza-Rosales P, Guzmán-Delgado NE, Ramírez-Montoya H, Martínez-Silva MG, et al. Expression of NK cell surface receptors in breast cancer tissue as predictors of resistance to antineoplastic treatment. Technol Cancer Res Treat. 2018;17:1533033818764499. 29558872 10.1177/1533033818764499 PMC5882046

[174] Mamessier E, Sylvain A, Thibult ML, Houvenaeghel G, Jacquemier J, Castellano R, et al. Human breast cancer cells enhance self tolerance by promoting evasion from NK cell antitumor immunity. J Clin Investig. 2011;121:3609–22. 21841316 10.1172/JCI45816 PMC3171102

[175] Terrén I, Orrantia A, Vitallé J, Zenarruzabeitia O, Borrego F. NK cell metabolism and tumor microenvironment. Front Immunol. 2019;10:2278. 31616440 10.3389/fimmu.2019.02278 PMC6769035

[176] Cluxton CD, Spillane C, O'Toole SA, Sheils O, Gardiner CM, O'Leary JJ. Suppression of natural killer cell NKG2D and CD226 anti-tumour cascades by platelet cloaked cancer cells: implications for the metastatic cascade. PLoS ONE. 2019;14:e0211538. 30908480 10.1371/journal.pone.0211538 PMC6433214

[177] Close HJ, Stead LF, Nsengimana J, Reilly KA, Droop A, Wurdak H, et al. Expression profiling of single cells and patient cohorts identifies multiple immunosuppressive pathways and an altered NK cell phenotype in glioblastoma. Clin Exp Immunol. 2020;200:33–44. 31784984 10.1111/cei.13403 PMC7066386

[178] Park A, Lee Y, Kim MS, Kang YJ, Park YJ, Jung H, et al. Prostaglandin E2 secreted by thyroid cancer cells contributes to immune escape through the suppression of natural killer (NK) cell cytotoxicity and NK cell differentiation. Front Immunol. 2018;9:1859. 30140269 10.3389/fimmu.2018.01859 PMC6094168

[179] Zhang Y, Wallace DL, de Lara CM, Ghattas H, Asquith B, Worth A, et al. In vivo kinetics of human natural killer cells: the effects of ageing and acute and chronic viral infection. Immunology. 2007;121:258–65. 17346281 10.1111/j.1365-2567.2007.02573.x PMC2265941

[180] Gill S, Vasey AE, De Souza A, Baker J, Smith AT, Kohrt HE, et al. Rapid development of exhaustion and down-regulation of eomesodermin limit the antitumor activity of adoptively transferred murine natural killer cells. Blood. 2012;119:5758–68. 22544698 10.1182/blood-2012-03-415364 PMC3382935

[181] Ménard C, Blay JY, Borg C, Michiels S, Ghiringhelli F, Robert C, et al. Natural killer cell IFN-γ levels predict long-term survival with imatinib mesylate therapy in gastrointestinal stromal tumor–bearing patients. Cancer Res. 2009;69:3563–9. 19351841 10.1158/0008-5472.CAN-08-3807

[182] Pernot S, Terme M, Radosevic-Robin N, Castan F, Badoual C, Marcheteau E, et al. Infiltrating and peripheral immune cell analysis in advanced gastric cancer according to the Lauren classification and its prognostic significance. Gastric Cancer. 2020;23:73–81. 31267360 10.1007/s10120-019-00983-3

[183] Semeraro M, Rusakiewicz S, Minard-Colin V, Delahaye NF, Enot D, Vély F, et al. Clinical impact of the NKp30/B7-H6 axis in high-risk neuroblastoma patients. Sci Transl Med. 2015;7:283. 10.1126/scitranslmed.aaa2327 25877893

[184] Xiao L, Cen D, Gan H, Sun Y, Huang N, Xiong H, et al. Adoptive transfer of NKG2D CAR mRNA-engineered natural killer cells in colorectal cancer patients. Mol Ther. 2019;27:1114–25. 30962163 10.1016/j.ymthe.2019.03.011 PMC6554529

[185] Kremer V, Ligtenberg MA, Zendehdel R, Seitz C, Duivenvoorden A, Wennerberg E, et al. Genetic engineering of human NK cells to express CXCR2 improves migration to renal cell carcinoma. J Immunother Cancer. 2017;5:73. 28923105 10.1186/s40425-017-0275-9 PMC5604543

[186] Ng YY, Tay JCK, Wang S. CXCR1 expression to improve anti-cancer efficacy of intravenously injected CAR-NK cells in mice with peritoneal xenografts. Mol Ther Oncolytics. 2020;16:75–85. 31970285 10.1016/j.omto.2019.12.006 PMC6965500

[187] Bonanni V, Antonangeli F, Santoni A, Bernardini G. Targeting of CXCR3 improves anti-myeloma efficacy of adoptively transferred activated natural killer cells. J Immunother Cancer. 2019;7:290. 31699153 10.1186/s40425-019-0751-5 PMC6839099

[188] Lee J, Kang TH, Yoo W, Choi H, Jo S, Kong K, et al. An antibody designed to improve adoptive NK-cell therapy inhibits pancreatic cancer progression in a murine model. Cancer Immunol Res. 2019;7:219–29. 30514792 10.1158/2326-6066.CIR-18-0317

[189] Melaiu O, Lucarini V, Cifaldi L, Fruci D. Influence of the tumor microenvironment on NK cell function in solid tumors. Front Immunol. 2020;10:3038. 32038612 10.3389/fimmu.2019.03038 PMC6985149

[190] Trotta R, Dal Col J, Yu J, Ciarlariello D, Thomas B, Zhang X, et al. TGF-β utilizes SMAD3 to inhibit CD16-mediated IFN-γ production and antibody-dependent cellular cytotoxicity in human NK cells. J Immunol. 2008;181:3784–92. 18768831 10.4049/jimmunol.181.6.3784 PMC2924753

[191] Friese MA, Wischhusen J, Wick W, Weiler M, Eisele G, Steinle A, et al. RNA interference targeting transforming growth factor-β enhances NKG2D-mediated antiglioma immune response, inhibits glioma cell migration and invasiveness, and abrogates tumorigenicity in vivo. Cancer Res. 2004;64:7596–603. 15492287 10.1158/0008-5472.CAN-04-1627

[192] Crane CA, Han SJ, Barry JJ, Ahn BJ, Lanier LL, Parsa AT. TGF-beta downregulates the activating receptor NKG2D on NK cells and CD8+ T cells in glioma patients. Neurooncology. 2010;12:7–13. 10.1093/neuonc/nop009 PMC2940557 20150362

[193] Kopp H-G, Placke T, Salih HR. Platelet-derived transforming growth factor-β down-regulates NKG2D thereby inhibiting natural killer cell antitumor reactivity. Cancer Res. 2009;69:7775–83. 19738039 10.1158/0008-5472.CAN-09-2123

[194] Yvon ES, Burga R, Powell A, Cruz CR, Fernandes R, Barese C, et al. Cord blood natural killer cells expressing a dominant negative TGF-β receptor: implications for adoptive immunotherapy for glioblastoma. Cytotherapy. 2017;19:408–18. 28109751 10.1016/j.jcyt.2016.12.005

[195] Rouce RH, Shaim H, Sekine T, Weber G, Ballard B, Ku S, et al. The TGF-β/SMAD pathway is an important mechanism for NK cell immune evasion in childhood B-acute lymphoblastic leukemia. Leukemia. 2016;30:800–11. 26621337 10.1038/leu.2015.327 PMC4823160

[196] Shaim H, Shanley M, Basar R, Daher M, Gumin J, Zamler DB, et al. Targeting the αv integrin/TGF-β axis improves natural killer cell function against glioblastoma stem cells. J Clin Investig. 2021;131:e142116. 34138753 10.1172/JCI142116 PMC8279586

[197] Chambers AM, Wang J, Lupo KB, Yu H, Atallah Lanman NM, Matosevic S. Adenosinergic signaling alters natural killer cell functional responses. Front Immunol. 2018;9:2533. 30425720 10.3389/fimmu.2018.02533 PMC6218627

[198] Antonioli L, Blandizzi C, Pacher P, Haskó G. Immunity, inflammation and cancer: a leading role for adenosine. Nat Rev Cancer. 2013;13:842–57. 24226193 10.1038/nrc3613

[199] Ryzhov S, Novitskiy SV, Goldstein AE, Biktasova A, Blackburn MR, Biaggioni I, et al. Adenosinergic regulation of the expansion and immunosuppressive activity of CD11b+Gr1+ cells. J Immunol. 2011;187:6120–9. 22039302 10.4049/jimmunol.1101225 PMC3221925

[200] Sun X, Wu Y, Gao W, Enjyoji K, Csizmadia E, Müller CE, et al. CD39/ENTPD1 expression by CD4+Foxp3+ regulatory T cells promotes hepatic metastatic tumor growth in mice. Gastroenterology. 2010;139:1030–40. 20546740 10.1053/j.gastro.2010.05.007 PMC2930043

[201] Augustin RC, Leone RD, Naing A, Fong L, Bao R, Luke JJ. Next steps for clinical translation of adenosine pathway inhibition in cancer immunotherapy. J Immunother Cancer. 2022;10:e004089. 35135866 10.1136/jitc-2021-004089 PMC8830302

[202] Young A, Ngiow SF, Barkauskas DS, Sult E, Hay C, Blake SJ, et al. Co-inhibition of CD73 and A2AR adenosine signaling improves anti-tumor immune responses. Cancer Cell. 2016;30:391–403. 27622332 10.1016/j.ccell.2016.06.025

[203] Young A, Ngiow SF, Gao Y, Patch AM, Barkauskas DS, Messaoudene M, et al. A2AR adenosine signaling suppresses natural killer cell maturation in the tumor microenvironment. Cancer Res. 2018;78:1003–16. 29229601 10.1158/0008-5472.CAN-17-2826

[204] Giuffrida L, Sek K, Henderson MA, Lai J, Chen A, Meyran D, et al. CRISPR/Cas9 mediated deletion of the adenosine A2A receptor enhances CAR T cell efficacy. Nat Commun. 2021;12:3236. 34050151 10.1038/s41467-021-23331-5 PMC8163771

[205] Stojanovic A, Fiegler N, Brunner-Weinzierl M, Cerwenka A. CTLA-4 is expressed by activated mouse NK cells and inhibits NK cell IFN-γ production in response to mature dendritic cells. J Immunol. 2014;192:4184–91. 24688023 10.4049/jimmunol.1302091

[206] Davis Z, Felices M, Lenvik T, Badal S, Walker JT, Hinderlie P, et al. Low-density PD-1 expression on resting human natural killer cells is functional and upregulated after transplantation. Blood Adv. 2021;5:1069–80. 33599743 10.1182/bloodadvances.2019001110 PMC7903227

[207] Sanseviero E, O'Brien EM, Karras JR, Shabaneh TB, Aksoy BA, Xu W, et al. Anti–CTLA-4 activates intratumoral NK cells and combined with IL15/IL15Rα complexes enhances tumor control. Cancer Immunol Res. 2019;7:1371–80. 31239316 10.1158/2326-6066.CIR-18-0386 PMC6956982

[208] Hsu J, Hodgins JJ, Marathe M, Nicolai CJ, Bourgeois-Daigneault MC, Trevino TN, et al. Contribution of NK cells to immunotherapy mediated by PD-1/PD-L1 blockade. J Clin Investig. 2018;128:4654–68. 30198904 10.1172/JCI99317 PMC6159991

[209] Creelan BC, Antonia SJ. The NKG2A immune checkpoint—a new direction in cancer immunotherapy. Nat Rev Clin Oncol. 2019;16:277–8. 30824813 10.1038/s41571-019-0182-8

[210] Laskowski TJ, Biederstädt A, Rezvani K. Natural killer cells in antitumour adoptive cell immunotherapy. Nat Rev Cancer. 2022;22:557–75. 35879429 10.1038/s41568-022-00491-0 PMC9309992

[211] da Silva IP, Gallois A, Jimenez-Baranda S, Khan S, Anderson AC, Kuchroo VK, et al. Reversal of NK-cell exhaustion in advanced melanoma by Tim-3 blockade. Cancer Immunol Res. 2014;2:410–22. 24795354 10.1158/2326-6066.CIR-13-0171 PMC4046278

[212] Zhang Q, Bi J, Zheng X, Chen Y, Wang H, Wu W, et al. Blockade of the checkpoint receptor TIGIT prevents NK cell exhaustion and elicits potent anti-tumor immunity. Nat Immunol. 2018;19:723–32. 29915296 10.1038/s41590-018-0132-0

[213] André P, Denis C, Soulas C, Bourbon-Caillet C, Lopez J, Arnoux T, et al. Anti-NKG2A mAb is a checkpoint inhibitor that promotes anti-tumor immunity by unleashing both T and NK cells. Cell. 2018;175:1731–43. 30503213 10.1016/j.cell.2018.10.014 PMC6292840

[214] Miller JS, Soignier Y, Panoskaltsis-Mortari A, McNearney SA, Yun GH, Fautsch SK, et al. Successful adoptive transfer and in vivo expansion of human haploidentical NK cells in patients with cancer. Blood. 2005;105:3051–7. 15632206 10.1182/blood-2004-07-2974

[215] Bachanova V, Cooley S, Defor TE, Verneris MR, Zhang B, McKenna DH, et al. Clearance of acute myeloid leukemia by haploidentical natural killer cells is improved using IL-2 diphtheria toxin fusion protein. Blood. 2014;123:3855–63. 24719405 10.1182/blood-2013-10-532531 PMC4064329

[216] Liu E, Marin D, Banerjee P, Macapinlac HA, Thompson P, Basar R, et al. Use of CAR-transduced natural killer cells in CD19-positive lymphoid tumors. N Engl J Med. 2020;382:545–53. 32023374 10.1056/NEJMoa1910607 PMC7101242

[217] Marin D, Li Y, Basar R, Rafei H, Daher M, Dou J, et al. Safety, efficacy and determinants of response of allogeneic CD19-specific CAR-NK cells in CD19+ B cell tumors: a phase 1/2 trial. Nat Med. 2024;30:772–84. 38238616 10.1038/s41591-023-02785-8 PMC10957466

[218] Daher M, Basar R, Gokdemir E, Baran N, Uprety N, Nunez Cortes AK, et al. Targeting a cytokine checkpoint enhances the fitness of armored cord blood CAR-NK cells. Blood. 2021;137:624–36. 32902645 10.1182/blood.2020007748 PMC7869185

[219] Mansour AG, Teng KY, Li Z, Zhu Z, Chen H, Tian L, et al. Off-the-shelf CAR-engineered natural killer cells targeting FLT3 enhance killing of acute myeloid leukemia. Blood Adv. 2023;7:6225–39. 37379267 10.1182/bloodadvances.2022007405 PMC10582841

[220] Teng K-Y, Mansour AG, Zhu Z, Li Z, Tian L, Ma S, et al. Off-the-shelf prostate stem cell antigen-directed chimeric antigen receptor natural killer cell therapy to treat pancreatic cancer. Gastroenterology. 2022;162:1319–33. 34999097 10.1053/j.gastro.2021.12.281 PMC8963130

[221] Cerwenka A, Lanier LL. Natural killer cell memory in infection, inflammation and cancer. Nat Rev Immunol. 2016;16:112–23. 26806484 10.1038/nri.2015.9

[222] Farber DL, Netea MG, Radbruch A, Rajewsky K, Zinkernagel RM. Immunological memory: lessons from the past and a look to the future. Nat Rev Immunol. 2016;16:124–8. 26831526 10.1038/nri.2016.13

[223] Shapiro RM, Birch GC, Hu G, Vergara Cadavid J, Nikiforow S, Baginska J, et al. Expansion, persistence, and efficacy of donor memory-like NK cells infused for posttransplant relapse. J Clin Investig. 2022;132:e154334. 35349491 10.1172/JCI154334 PMC9151697

[224] Romee R, Rosario M, Berrien-Elliott MM, Wagner JA, Jewell BA, Schappe T, et al. Cytokine-induced memory-like natural killer cells exhibit enhanced responses against myeloid leukemia. Sci Transl Med. 2016;8:357. 10.1126/scitranslmed.aaf2341 PMC5436500 27655849

[225] Bednarski JJ, Zimmerman C, Berrien-Elliott MM, Foltz JA, Becker-Hapak M, Neal CC, et al. Donor memory-like NK cells persist and induce remissions in pediatric patients with relapsed AML after transplant. Blood. 2022;139:1670–83. 34871371 10.1182/blood.2021013972 PMC8931511

[226] Dong H, Ham JD, Hu G, Xie G, Vergara J, Liang Y, et al. Memory-like NK cells armed with a neoepitope-specific CAR exhibit potent activity against NPM1 mutated acute myeloid leukemia. Proc Natl Acad Sci USA. 2022;119:e2122379119. 35696582 10.1073/pnas.2122379119 PMC9231490

[227] Gasser S, Orsulic S, Brown EJ, Raulet DH. The DNA damage pathway regulates innate immune system ligands of the NKG2D receptor. Nature. 2005;436:1186–90. 15995699 10.1038/nature03884 PMC1352168

[228] Leivas A, Valeri A, Córdoba L, García-Ortiz A, Ortiz A, Sánchez-Vega L, et al. NKG2D-CAR-transduced natural killer cells efficiently target multiple myeloma. Blood Cancer J. 2021;11:146. 34392311 10.1038/s41408-021-00537-w PMC8364555

[229] Eisenberg V, Shamalov K, Meir S, Hoogi S, Sarkar R, Pinker S, et al. Targeting multiple tumors using T-cells engineered to express a natural cytotoxicity receptor 2-based chimeric receptor. Front Immunol. 2017;8:1212. 29085357 10.3389/fimmu.2017.01212 PMC5649149

[230] Tal Y, Yaakobi S, Horovitz-Fried M, Safyon E, Rosental B, Porgador A, et al. An NCR1-based chimeric receptor endows T-cells with multiple anti-tumor specificities. Oncotarget. 2014;5:10949–58. 25431955 10.18632/oncotarget.1919 PMC4279421

[231] Li Y, Hermanson DL, Moriarity BS, Kaufman DS. Human iPSC-derived natural killer cells engineered with chimeric antigen receptors enhance anti-tumor activity. Cell Stem Cell. 2018;23:181–92. 30082067 10.1016/j.stem.2018.06.002 PMC6084450

[232] Lanier LL, Phillips JH. NK cell recognition of major histocompatibility complex class I molecules. Semin Immunol. 1995;7:75–82. 7579197 10.1006/smim.1995.0011

[233] Barrow AD, Martin CJ, Colonna M. The natural cytotoxicity receptors in health and disease. Front Immunol. 2019;10:909. 31134055 10.3389/fimmu.2019.00909 PMC6514059

[234] Siegler EL, Zhu Y, Wang P, Yang L. Off-the-shelf CAR-NK cells for cancer immunotherapy. Cell Stem Cell. 2018;23:160–1. 30075127 10.1016/j.stem.2018.07.007

[235] Gong JH, Maki G, Klingemann HG. Characterization of a human cell line (NK-92) with phenotypical and functional characteristics of activated natural killer cells. Leukemia. 1994;8:652–8. 8152260

[236] Tang X, Yang L, Li Z, Nalin AP, Dai H, Xu T, et al. First-in-man clinical trial of CAR NK-92 cells: safety test of CD33-CAR NK-92 cells in patients with relapsed and refractory acute myeloid leukemia. Am J Cancer Res. 2018;8:1083–9. 30034945 PMC6048396

[237] Hermanson DL, Kaufman DS. Utilizing chimeric antigen receptors to direct natural killer cell activity. Front Immunol. 2015;6:195. 25972867 10.3389/fimmu.2015.00195 PMC4412125

[238] Drexler H, Matsuo Y. Malignant hematopoietic cell lines: in vitro models for the study of natural killer cell leukemia–lymphoma. Leukemia. 2000;14:777–82. 10803505 10.1038/sj.leu.2401778

[239] Freud AG, Mundy-Bosse BL, Yu J, Caligiuri MA. The broad spectrum of human natural killer cell diversity. Immunity. 2017;47:820–33. 29166586 10.1016/j.immuni.2017.10.008 PMC5728700

[240] Lamers-Kok N, Panella D, Georgoudaki AM, Liu H, Özkazanc D, Kučerová L, et al. Natural killer cells in clinical development as non-engineered, engineered, and combination therapies. J Hematol Oncol. 2022;15:164. 36348457 10.1186/s13045-022-01382-5 PMC9644572

[241] Luevano M, Daryouzeh M, Alnabhan R, Querol S, Khakoo S, Madrigal A, et al. The unique profile of cord blood natural killer cells balances incomplete maturation and effective killing function upon activation. Hum Immunol. 2012;73:248–57. 22234167 10.1016/j.humimm.2011.12.015

[242] Dalle J-H, Menezes J, Wagner E, Blagdon M, Champagne J, Champagne MA, et al. Characterization of cord blood natural killer cells: implications for transplantation and neonatal infections. Pediatr Res. 2005;57:649–55. 15718362 10.1203/01.PDR.0000156501.55431.20

[243] Dolstra H, Roeven M, Spanholtz J, Hangalapura BN, Tordoir M, Maas F, et al. Successful transfer of umbilical cord blood CD34+ hematopoietic stem and progenitor-derived NK cells in older acute myeloid leukemia patients. Clin Cancer Res. 2017;23:4107–18. 28280089 10.1158/1078-0432.CCR-16-2981

[244] Takahashi K, Yamanaka S. Induction of pluripotent stem cells from mouse embryonic and adult fibroblast cultures by defined factors. Cell. 2006;126:663–76. 16904174 10.1016/j.cell.2006.07.024

[245] Zhu H, Blum RH, Bjordahl R, Gaidarova S, Rogers P, Lee TT, et al. Pluripotent stem cell–derived NK cells with high-affinity noncleavable CD16a mediate improved antitumor activity. Blood. 2020;135:399–410. 31856277 10.1182/blood.2019000621 PMC7005364

[246] Goodridge JP, Mahmood S, Zhu H, Gaidarova S, Blum R, Bjordahl R, et al. FT596: translation of first-of-kind multi-antigen targeted off-the-shelf CAR-NK cell with engineered persistence for the treatment of B cell malignancies. Blood. 2019;134:301–301.

[247] Bjordahl R, Gaidarova S, Woan K, Cichocki F, Bonello G, Robinson M, et al. FT538: preclinical development of an off-the-shelf adoptive NK cell immunotherapy with targeted disruption of CD38 to prevent anti-CD38 antibody-mediated fratricide and enhance ADCC in multiple myeloma when combined with daratumumab. Blood. 2019;134:133–133.

[248] Myers JA, Miller JS. Exploring the NK cell platform for cancer immunotherapy. Nat Rev Clin Oncol. 2021;18:85–100. 32934330 10.1038/s41571-020-0426-7 PMC8316981

[249] Zhu H, Blum RH, Bernareggi D, Ask EH, Wu Z, Hoel HJ, et al. Metabolic reprograming via deletion of CISH in human iPSC-derived NK cells promotes in vivo persistence and enhances anti-tumor activity. Cell Stem Cell. 2020;27:224–37. 32531207 10.1016/j.stem.2020.05.008 PMC7415618

[250] Sayitoglu EC, Georgoudaki AM, Chrobok M, Ozkazanc D, Josey BJ, Arif M, et al. Boosting natural killer cell-mediated targeting of sarcoma through DNAM-1 and NKG2D. Front Immunol. 2020;11:40. 32082316 10.3389/fimmu.2020.00040 PMC7001093

[251] Peng L, Renauer PA, Sferruzza G, Yang L, Zou, Y, Fang Z, et al. In vivo AAV–SB-CRISPR screens of tumor-infiltrating primary NK cells identify genetic checkpoints of CAR-NK therapy. Nat Biotechnol. 2024. 10.1038/s41587-024-02282-4. 10.1038/s41587-024-02282-4 PMC11668911 38918616

[252] Li Y, Basar R, Wang G, Liu E, Moyes JS, Li L, et al. KIR-based inhibitory CARs overcome CAR-NK cell trogocytosis-mediated fratricide and tumor escape. Nat Med. 2022;28:2133–44. 36175679 10.1038/s41591-022-02003-x PMC9942695

[253] Hsi ED, Steinle R, Balasa B, Szmania S, Draksharapu A, Shum BP, et al. CS1, a potential new therapeutic antibody target for the treatment of multiple myeloma. Clin Cancer Res J Am Assoc Cancer Res. 2008;14:2775–84. 10.1158/1078-0432.CCR-07-4246 PMC4433038 18451245

[254] Park JH, Rivière I, Gonen M, Wang X, Sénéchal B, Curran KJ, et al. Long-term follow-up of CD19 CAR therapy in acute lymphoblastic leukemia. N Engl J Med. 2018;378:449–59. 29385376 10.1056/NEJMoa1709919 PMC6637939

[255] Brudno JN, Somerville RP, Shi V, Rose JJ, Halverson DC, Fowler DH, et al. Allogeneic T cells that express an anti-CD19 chimeric antigen receptor induce remissions of B-cell malignancies that progress after allogeneic hematopoietic stem-cell transplantation without causing graft-versus-host disease. J Clin Oncol J Am Soc Clin Oncol. 2016;34:1112–21. 10.1200/JCO.2015.64.5929 PMC4872017 26811520

[256] Mallapaty S. Cutting-edge CAR-T cancer therapy is now made in India - at one-tenth the cost. Nature. 2024;627:709–10. 38514877 10.1038/d41586-024-00809-y

[257] Benjamin R, Graham C, Yallop D, Jozwik A, Mirci-Danicar OC, Lucchini G, et al. Genome-edited, donor-derived allogeneic anti-CD19 chimeric antigen receptor T cells in paediatric and adult B-cell acute lymphoblastic leukaemia: results of two phase 1 studies. Lancet. 2020;396:1885–94. 33308471 10.1016/S0140-6736(20)32334-5 PMC11773457

[258] Benjamin R, Jain N, Maus MV, Boissel N, Graham C, Jozwik A, et al. UCART19, a first-in-class allogeneic anti-CD19 chimeric antigen receptor T-cell therapy for adults with relapsed or refractory B-cell acute lymphoblastic leukaemia (CALM): a phase 1, dose-escalation trial. Lancet Haematol. 2022;9:e833–e843. 36228643 10.1016/S2352-3026(22)00245-9 PMC11575699

[259] Mailankody S, Matous JV, Chhabra S, Liedtke M, Sidana S, Oluwole OO, et al. Allogeneic BCMA-targeting CAR T cells in relapsed/refractory multiple myeloma: phase 1 UNIVERSAL trial interim results. Nat Med. 2023;29:422–9. 36690811 10.1038/s41591-022-02182-7

[260] Chiesa R, Georgiadis C, Syed F, Zhan H, Etuk A, Gkazi SA, et al. Base-edited CAR7 T cells for relapsed T-cell acute lymphoblastic leukemia. N Engl J Med. 2023;389:899–910. 37314354 10.1056/NEJMoa2300709

[261] Liu E, Tong Y, Dotti G, Shaim H, Savoldo B, Mukherjee M, et al. Cord blood NK cells engineered to express IL-15 and a CD19-targeted CAR show long-term persistence and potent antitumor activity. Leukemia. 2018;32:520–31. 28725044 10.1038/leu.2017.226 PMC6063081

[262] Duygu B, Olieslagers TI, Groeneweg M, Voorter CEM, Wieten LHLA, et al. Molecules as immune checkpoints for NK cell alloreactivity and anti-viral immunity in kidney transplantation. Front Immunol. 2021;12:680480. 34295330 10.3389/fimmu.2021.680480 PMC8290519

[263] Poirot L, Philip B, Schiffer-Mannioui C, Le Clerre D, Chion-Sotinel I, Derniame S, et al. Multiplex genome-edited T-cell manufacturing platform for ‘off-the-shelf’ adoptive T-cell immunotherapies. Cancer Res. 2015;75:3853–64. 26183927 10.1158/0008-5472.CAN-14-3321

[264] Jasinski-Bergner S, Eckstein M, Taubert H, Wach S, Fiebig C, Strick R, et al. The human leukocyte antigen G as an immune escape mechanism and novel therapeutic target in urological tumors. Front Immunol. 2022;13:811200. 35185904 10.3389/fimmu.2022.811200 PMC8855320

[265] Neelapu SS, Locke FL, Bartlett NL, Lekakis LJ, Miklos DB, Jacobson CA, et al. Axicabtagene ciloleucel CAR T-cell therapy in refractory large B-cell lymphoma. N Engl J Med. 2017;377:2531–44. 29226797 10.1056/NEJMoa1707447 PMC5882485

[266] Shi J, Tricot G, Szmania S, Rosen N, Garg TK, Malaviarachchi PA, et al. Infusion of haplo‐identical killer immunoglobulin‐like receptor ligand mismatched NK cells for relapsed myeloma in the setting of autologous stem cell transplantation. Br J Haematol. 2008;143:641–53. 18950462 10.1111/j.1365-2141.2008.07340.x PMC3602915

[267] Björklund AT, Carlsten M, Sohlberg E, Liu LL, Clancy T, Karimi M, et al. Complete remission with reduction of high-risk clones following haploidentical NK-cell therapy against MDS and AML. Clin Cancer Res. 2018;24:1834–44. 29444931 10.1158/1078-0432.CCR-17-3196

[268] Alnaggar M, Lin M, Mesmar A, Liang S, Qaid A, Xu K, et al. Allogenic natural killer cell immunotherapy combined with irreversible electroporation for stage IV hepatocellular carcinoma: survival outcome. Cell Physiol Biochem. 2018;48:1882–93. 30092590 10.1159/000492509

[269] Zhang X, Guo Y, Ji Y, Gao Y, Zhang M, Liu Y, et al. Cytokine release syndrome after modified CAR-NK therapy in an advanced non-small cell lung cancer patient: a case report. Cell Transplant. 2022;31:096368972210942. 10.1177/09636897221094244 PMC9073124 35506155

[270] Oei V, Siernicka M, Graczyk-Jarzynka A, Hoel HJ, Yang W, Palacios D, et al. Intrinsic functional potential of NK-cell subsets constrains retargeting driven by chimeric antigen receptors. Cancer Immunol Res. 2018;6:467–80. 29459477 10.1158/2326-6066.CIR-17-0207

[271] Touat M, Li YY, Boynton AN, Spurr LF, Iorgulescu JB, Bohrson CL, et al. Mechanisms and therapeutic implications of hypermutation in gliomas. Nature. 2020;580:517–23. 32322066 10.1038/s41586-020-2209-9 PMC8235024

[272] Ishigami S, Natsugoe S, Tokuda K, Nakajo A, Che X, Iwashige H, et al. Prognostic value of intratumoral natural killer cells in gastric carcinoma. Cancer. 2000;88:577–83. 10649250

[273] Villegas FR, Coca S, Villarrubia VG, Jiménez R, Chillón MJ, Jareño J, et al. Prognostic significance of tumor infiltrating natural killer cells subset CD57 in patients with squamous cell lung cancer. Lung Cancer. 2002;35:23–28. 11750709 10.1016/s0169-5002(01)00292-6

[274] Carlsten M, Childs RW. Genetic manipulation of NK cells for cancer immunotherapy: techniques and clinical implications. Front Immunol. 2015;6:266. 26113846 10.3389/fimmu.2015.00266 PMC4462109

[275] Barrett DM, Singh N, Porter DL, Grupp SA, June CH. Chimeric antigen receptor therapy for cancer. Annu Rev Med. 2014;65:333–47. 24274181 10.1146/annurev-med-060512-150254 PMC4120077

[276] Streltsova MA, Barsov E, Erokhina SA, Kovalenko EI. Retroviral gene transfer into primary human NK cells activated by IL-2 and K562 feeder cells expressing membrane-bound IL-21. J Immunol Methods. 2017;450:90–94. 28802832 10.1016/j.jim.2017.08.003

[277] Cavazza A, Moiani A, Mavilio F. Mechanisms of retroviral integration and mutagenesis. Hum Gene Ther. 2013;24:119–31. 23330935 10.1089/hum.2012.203

[278] Hacein-Bey-Abina S, Von Kalle C, Schmidt M, McCormack MP, Wulffraat N, Leboulch P, et al. LMO2-associated clonal T cell proliferation in two patients after gene therapy for SCID-X1. Science. 2003;302:415–9. 14564000 10.1126/science.1088547

[279] Hacein-Bey-Abina S, Garrigue A, Wang GP, Soulier J, Lim A, Morillon E, et al. Insertional oncogenesis in 4 patients after retrovirus-mediated gene therapy of SCID-X1. J Clin Investig. 2008;118:3132–42. 18688285 10.1172/JCI35700 PMC2496963

[280] Kustikova O, Fehse B, Modlich U, Yang M, Düllmann J, Kamino K, et al. Clonal dominance of hematopoietic stem cells triggered by retroviral gene marking. Science. 2005;308:1171–4. 15905401 10.1126/science.1105063

[281] Sutlu T, Nyström S, Gilljam M, Stellan B, Applequist SE, Alici E. Inhibition of intracellular antiviral defense mechanisms augments lentiviral transduction of human natural killer cells: implications for gene therapy. Hum Gene Ther. 2012;23:1090–1100. 22779406 10.1089/hum.2012.080 PMC3472531

[282] Micucci F, Zingoni A, Piccoli M, Frati L, Santoni A, Galandrini R. High-efficient lentiviral vector-mediated gene transfer into primary human NK cells. Exp Hematol. 2006;34:1344–52. 16982327 10.1016/j.exphem.2006.06.001

[283] Tran J, Kung SKP. Lentiviral vectors mediate stable and efficient gene delivery into primary murine natural killer cells. Mol Ther J Am Soc Gene Ther. 2007;15:1331–9. 10.1038/sj.mt.6300184 17505478

[284] Allan D, Chakraborty M, Waller GC, Hochman MJ, Poolcharoen A, Reger RN, et al. Systematic improvements in lentiviral transduction of primary human natural killer cells undergoing ex vivo expansion. Mol Ther Methods Clin Dev. 2021;20:559–71. 33665226 10.1016/j.omtm.2021.01.008 PMC7890427

[285] Kruschinski A, Moosmann A, Poschke I, Norell H, Chmielewski M, Seliger B, et al. Engineering antigen-specific primary human NK cells against HER-2 positive carcinomas. Proc Natl Acad Sci USA. 2008;105:17481–6. 18987320 10.1073/pnas.0804788105 PMC2582261

[286] Pfefferle A, Jacobs B, Haroun-Izquierdo A, Kveberg L, Sohlberg E, Malmberg KJ. Deciphering natural killer cell homeostasis. Front Immunol. 2020;11:812. 32477340 10.3389/fimmu.2020.00812 PMC7235169

[287] Gauthier J, Bezerra ED, Hirayama AV, Fiorenza S, Sheih A, Chou CK, et al. Factors associated with outcomes after a second CD19-targeted CAR T-cell infusion for refractory B-cell malignancies. Blood. 2021;137:323–35. 32967009 10.1182/blood.2020006770 PMC7819764

[288] Patel K, Bachanova V, Goodman AM, Pagel JM, Griffis K, Anderson M, et al. Phase I study of FT516, an off-the-shelf iPSC-derived NK cell therapy, in combination with rituximab in patients with relapsed/refractory B-cell lymphoma. Blood. 2021;138:3873–3873.

[289] Verdun N, Marks P. Secondary cancers after chimeric antigen receptor T-cell therapy. N Engl J Med. 2024;390:584–6. 38265704 10.1056/NEJMp2400209

[290] Willyard C. Do cutting-edge CAR-T-cell therapies cause cancer? What the data say. Nature. 2024;629:22–24. 38689052 10.1038/d41586-024-01215-0

[291] Müller F, Taubmann J, Bucci L, Wilhelm A, Bergmann C, Völkl S, et al. CD19 CAR T-cell therapy in autoimmune disease—a case series with follow-up. N Engl J Med. 2024;390:687–700. 38381673 10.1056/NEJMoa2308917

[292] Carvalho T. First two patients receive CAR T cell therapy for HIV. Nat Med. 2023;29:1290–1. 37161063 10.1038/d41591-023-00042-6

[293] Tiberghien P, Ferrand C, Lioure B, Milpied N, Angonin R, Deconinck E, et al. Administration of herpes simplex-thymidine kinase-expressing donor T cells with a T-cell-depleted allogeneic marrow graft. Blood. 2001;97:63–72. 11133743 10.1182/blood.v97.1.63

[294] Shy BR, Vykunta VS, Ha A, Talbot A, Roth TL, Nguyen DN, et al. High-yield genome engineering in primary cells using a hybrid ssDNA repair template and small-molecule cocktails. Nat Biotechnol. 2023;41:521–31. 36008610 10.1038/s41587-022-01418-8 PMC10065198

[295] Magnani CF, Gaipa G, Lussana F, Belotti D, Gritti G, Napolitano S, et al. Sleeping Beauty-engineered CAR T cells achieve antileukemic activity without severe toxicities. J Clin Investig. 2020;130:6021–33. 32780725 10.1172/JCI138473 PMC7598053

[296] Kebriaei P, Singh H, Huls MH, Figliola MJ, Bassett R, Olivares S, et al. Phase I trials using Sleeping Beauty to generate CD19-specific CAR T cells. J Clin Investig. 2016;126:3363–76. 27482888 10.1172/JCI86721 PMC5004935

[297] Zhang Y, Zhang Z, Ding Y, Fang Y, Wang P, Chu W, et al. Phase I clinical trial of EGFR-specific CAR-T cells generated by the piggyBac transposon system in advanced relapsed/refractory non-small cell lung cancer patients. J Cancer Res Clin Oncol. 2021;147:3725–34. 34032893 10.1007/s00432-021-03613-7 PMC11801842

[298] Balke-Want H, Keerthi V, Cadinanos-Garai A, Fowler C, Gkitsas N, Brown AK, et al. Non-viral chimeric antigen receptor (CAR) T cells going viral. Immuno Oncol Technol. 2023;18:100375. 10.1016/j.iotech.2023.100375 PMC10139995 37124148

[299] Geurts AM, Collier LS, Geurts JL, Oseth LL, Bell ML, Mu D, et al. Gene mutations and genomic rearrangements in the mouse as a result of transposon mobilization from chromosomal concatemers. PLoS Genet. 2006;2:e156. 17009875 10.1371/journal.pgen.0020156 PMC1584263

[300] Bachanova V, Ghobadi A, Patel K, Park JH, Flinn IW, Shah P, et al. Safety and efficacy of FT596, a first-in-class, multi-antigen targeted, off-the-shelf, iPSC-derived CD19 CAR NK cell therapy in relapsed/refractory B-cell lymphoma. Blood. 2021;138:823–823.

